# Breast Milk-Derived Extracellular Vesicles Enriched in Exosomes From Mothers With Type 1 Diabetes Contain Aberrant Levels of microRNAs

**DOI:** 10.3389/fimmu.2019.02543

**Published:** 2019-10-25

**Authors:** Aashiq H. Mirza, Simranjeet Kaur, Lotte B. Nielsen, Joachim Størling, Reza Yarani, Martin Roursgaard, Elisabeth R. Mathiesen, Peter Damm, Jens Svare, Henrik B. Mortensen, Flemming Pociot

**Affiliations:** ^1^Faculty of Health and Medical Sciences, University of Copenhagen, Copenhagen, Denmark; ^2^Department of Pediatrics E, Copenhagen Diabetes Research Center (CPH-DIRECT), Herlev and Gentofte Hospital, Herlev, Denmark; ^3^Steno Diabetes Center Copenhagen, Gentofte, Denmark; ^4^Faculty of Health Sciences, Institute of Public Health, CSS, University of Copenhagen, Copenhagen, Denmark; ^5^Department of Endocrinology, Rigshospitalet, Copenhagen, Denmark; ^6^Center for Pregnant Women With Diabetes, Rigshospitalet, Copenhagen, Denmark; ^7^Department of Obstetrics, Rigshospitalet, Copenhagen, Denmark; ^8^Department of Obstetrics, Herlev Hospital, Herlev, Denmark

**Keywords:** breast milk, exosomes, miRNAs, Type 1 diabetes, exomiRs

## Abstract

The breast milk plays a crucial role in shaping the initial intestinal microbiota and mucosal immunity of the infant. Interestingly, breastfeeding has proven to be protective against the early onset of immune-mediated diseases including type 1 diabetes. Studies have shown that exosomes from human breast milk are enriched in immune-modulating miRNAs suggesting that exosomal miRNAs (exomiRs) transferred to the infant could play a critical role in the development of the infant's immune system. We extracted exomiRs from breast milk of 52 lactating mothers (26 mothers with type 1 diabetes and 26 healthy mothers), to identify any differences in the exomiR content between the two groups. Small RNA-sequencing was performed to identify known and novel miRNAs in both groups. A total of 631 exomiRs were detected by small RNA sequencing including immune-related miRNAs such as hsa-let-7c, hsa-miR-21, hsa-miR-34a, hsa-miR-146b, and hsa-miR-200b. In addition, ~200 novel miRNAs were identified in both type 1 diabetes and control samples. Among the known miRNAs, nine exomiR's were found differentially expressed in mothers with type 1 diabetes compared to healthy mothers. The highly up-regulated miRNAs, hsa-miR-4497, and hsa-miR-3178, increased lipopolysaccharide-induced expression and secretion of tumor necrosis factor α (TNFα) in human monocytes. The up-regulated miRNA target genes were significantly enriched for longevity-regulating pathways and FoxO signaling. Our findings suggest a role of breast milk-derived exomiRs in modulating the infant's immune system.

## Introduction

Exosomes are small membranous nano-vesicles (30–150 nm) of endocytic origin, which are released into the extracellular milieu, including bio-fluids such as blood plasma, urine, semen, bronchoalveolar lavage fluid, saliva, and breast milk (1). Exosomes transport plethora of molecules including functional RNAs such as microRNAs (miRNAs) and proteins, some of which have been shown to modulate the immune system (2, 3). Recent studies have shown that breast milk derived exosomes and their cargo survive mimicked gastric and pancreatic digestion conditions similar to those of the infant's gut environment and exosomes can be taken up by the intestinal cells *in vitro* (4, 5).

A number of studies have demonstrated that breastfeeding has protective and positive effects for the infant and is also associated with a reduced risk for type 1 diabetes (6–9). Breastfeeding has also shown to be protective against other immune mediated diseases such as asthma and celiac disease (10–12). However, most of the studies investigating association between breastfeeding and development of type 1 diabetes and islet autoimmunity are retrospective and observational (8, 9).

Human breast milk stimulates the proliferation of a well-balanced and diverse microbiota in the infant and provides passive protective functions such as antibacterial peptides, lactoferrin, lysozyme, and components of the innate immune response (13). It contains high amounts of IgA, cytokines, antibodies, hormones, long-chain fatty acids, indigestible oligosaccharides, and exosomal miRNAs; all these factors stimulate the development of the infant's own immune system (13, 14). Exosomal miRNAs (exomiRs) packaged inside exosomes in human breast milk are transferred from the mother's milk to the infant via the digestive tract where they may play a critical role in the development of the infant's immune system (2–5, 15). Milk derived miRNAs also promote thymic regulatory T cell (Treg) maturation, thereby preventing Th2-mediated atopic sensitization and atopic effector responses (16). Highly significant amounts of immune-modulatory miRNAs known to play a role in thymic Treg differentiation (miR-155, miR-146a, miR-21) have been found in exosomes derived from human and bovine milk (3, 17).

In the present study, we investigated the exosomal transcriptome of human breast milk using small RNA sequencing to elucidate the distribution and expression profile of exomiRs in mothers with type 1 diabetes and healthy mothers. We aimed not only to identify miRNAs in breast milk, but also expand the number of known miRNAs in human breast milk with the identification of novel miRNAs. Pathway analysis of target genes associated with the known exomiRs highlights their potential immunomodulatory effects in the breastfed infants and notably ingeminates well-recognized nutritive, cognitive, and immunity-based benefits of the breastfeeding.

## Materials and Methods

### Ethics Statement

All women involved in the study gave their signed informed consent to participate. The study was approved by the Ethical Committee for the Capital Region, Denmark (H-4-2013-008).

### Sample Collection

Human breast milk samples (50–100 ml) were collected from 52 lactating mothers (26 mothers with type 1 diabetes and 26 healthy mothers) 4 weeks after delivery using a manual breast pump in a sterile bottle and kept refrigerated at 4°C and collected within 24 h. Before storing at −80°C, 50 ml milk sample was diluted with an equal volume of 1X PBS (pH7.4) and centrifuged at 300 x g for 10 min at 4°C to remove the cellular debris (18). All samples were stored at −80°C until processed. Blood glucose levels, HbA1c, insulin dosage and anthropometric data were recorded for all mothers. Inclusion criteria were healthy, normal birth-weight infants born at gestational age ≥37 weeks and continuous breastfeeding. Exclusion criteria included type 2 diabetes, smoking, and complications during delivery.

### Isolation and Characterization of Extracellular Vesicles Enriched in Exosomes

Extracellular vesicles enriched in exosomes were isolated by serial ultra-centrifugation method as previously described with minor modifications (1). Samples were centrifuged at 3000 X g for 10 min at 4°C followed by sequential filtrating of the supernatant through 1.2, 0.8, 0.45, and 0.2 μm filters (VWR®). Resultant filtrate was centrifuged at 16500 X g for 45 min at 4°C and supernatant was again filtered using 0.2 μm filter followed by centrifugation at 12000 X g for 70 min at 4°C to pellet exosomes. Exosome pellet was resuspended in 300 μl of 1X PBS. Details of exosome characterization based on exosomal surface markers, Transmission Electron Microscopy, and NanoSight analysis are described in Supplementary File 1 (Figure S1).

### Small RNA Sequencing

Total RNA from the isolated exosomes was used for library construction and subjected to single-end sequencing generating 10M reads/sample. The small RNAseq was performed using TruSeq Small RNA Library Preparation Kit (#RS-200-0048; Illumina, San Diego, CA, USA). All samples were sequenced by 50-bp single end reads (SE-50) on an Illumina HiSeq 2500 platform. In-house bioinformatics pipeline for mapping known miRNAs, novel miRNA discovery and differential expression analyses were applied. Full details of the methods are provided in Supplementary File 1. The small RNA sequencing data from this study is availabe at ArrayExpress with accession number E-MTAB-7336.

### Exosomal miRNA Expression Validation by RT-qPCR

Total RNA was purified from the isolated exosomes using miRNeasy kit (Qiagen) according to the manufacturer's instructions. Exosomal RNA was quantified and characterized by NanoDrop® Spectrophotometer (Thermo Scientific) and Bioanalyzer RNA nano chip (Agilent), respectively. Expression levels of miRNAs in the exosomes were validated using individual stem-loop quantitative RT-PCR (qRT-PCR) TaqMan microRNA Assays (Applied Biosystems) according to the manufacturer's instructions. We performed qPCR assays for 8 differentially expressed miRNAs (hsa-miR-4497, hsa-miR-133a, hsa-miR-1246, hsa-miR-1290, hsa-miR-518e-3p, hsa-miR-629–3p, and hsa-miR-200c-5p) for which advanced miRNA assays were available. We used a subset of both T1D and control samples (*n* = 4). hsa-let-7a-5p was used as control and all samples were tested in duplicates (Supplementary File 1, Figure S1). The expression levels of these miRNAs were also tested in the plasma of same mothers.

### THP-1 and CaCo-2 Cells, Transfection, and Experimentation

Human monocytic THP-1 cells were grown in RPMI1640 medium containing 4.5 g/L glucose and 10% FBS. Cells were passaged twice weekly. For experimentation, cells were differentiated into macrophages by exposure to 100 nM phorbol 12-myristate 13-acetate (PMA) for 3 days after which the cells were transfected with 30 nM negative control miRNA or hsa-miR-4497, hsa-miR-3178, hsa-miR-133a, and hsa-miR-1246 from Dharmacon (GE Healthcare) using Lipofectamine RNAiMAX (ThermoFisher Scientific) according to the manufacturer's instructions. Human epithelial CaCo-2 cell lines were grown according to standard media requirements from ATCC [EMEM + 10% FBS + Penicillin (100 U/ml) + Streptomycin (100 μgr/ml)]. Cells were seeded at a density of 5 × 10^4^ cells/cm^2^ on type I collagen pre-coated 24-well plates for the miRNA transfection and LPS stimulation. Cells were grown for 24 h until proliferative stage (~80% confluent) then transfected with 30 nM negative control miRNA, hsa-miR-4497, hsa-miR-3178, hsa-miR-133a, and hsa-miR-1246 from Dharmacon (GE Healthcare) using Lipofectamine RNAiMAX (ThermoFisher Scientific) according to the manufacturer's instructions. Two days after transfection, cells were left untreated or exposed to 5 ng/mL lipopolysaccharide (LPS) for 3 h. For determination of transfection efficiency, cells were also transfected with a fluorescent small RNA molecule (siGLO green). All RNA memetics were purchased from Dharmacon.

Total RNA was extracted by miRNeasy mini kit spin columns (Qiagen) and cDNA was synthesized by the iScript™ cDNA Synthesis Kit (Biorad). Gene expression was quantified by real-time PCR using TaqMan assays (Applied Biosystems) on a CFX384 C1000 Termalcycler (Biorad). Gene expression was normalized to that of β-actin (*ACTB*). The relative expression levels were calculated by the ΔΔCt method.

Cell viability was measured by the CytoTox-Fluor^TM^ cytotoxicity assay (Promega) according to the supplier's instructions. TNFα release into the culture medium was determined using a human TNFα ELISA kit (Invitrogen).

### Statistical Analysis

All statistical analyses were performed in R programming language. Pearson correlations were calculated for the differentially expressed miRNAs (log2CPM) and HbA1c (mmol/mol). The relationship between miRNA expression (log2CPM), HbA1c (mmol/mol) and insulin dose was analyzed using ANCOVA regression analysis.

## Results

### Study Participant's Characteristics

The study design, participant anthropometrics and measured clinical parameters are presented in Table 1. The measured clinical parameters included HbA1c, blood glucose levels and insulin dose. There were no significant differences between age and body weight of the mothers in the groups. There was the expected difference in gestational age and weight of infant due to type 1 diabetes pregnancy guidelines.

**Table 1:** Study design and clinical parameters.

**No. of individuals**	**T1D(26)**		**Control(26)**	
	**Range**	**Average**	**Range**	**Average**
Age (years)	24–41	32.19	24–40	31.81
Weight (kg)	56–91	74.77	55–109	76.52
BMI (kg/m^2^)	20–36	27.01	21–38	26.92
HbA1c (mmol/mol)	27–65	42.42	16–34	27.69
BG (mmol/l)	5–26	10.04	4–7	5.63
Insulin Dose (IU)	10–60	35.2	NA	NA
Gestational age at delivery (weeks)	37–39	38.1	37–41	40.1
Age of infant (Days) at sampling	36–50	34.4	28–57	37.0

*The table summarizes the study design and provides various clinical parameters. Total 52 lactating mothers (26 with type 1 diabetes and 26 healthy controls) were recruited for the study. BG, Blood Glucose*.

### Exosomes Characterization

A total of 100 ml breast milk yielded 28 μg exosomes based on the exosome protein estimation. Western blot results confirmed the presence of exosomal surface markers CD81, CD63, and HSP70 (Supplementary File 1, Figure S1A). The expression profile of differentially expressed miRNAs was confirmed by RT-qPCR (Supplementary File 1, Figure S1B). Transmission electron microscopy (TEM) demonstrated that exosomes isolated were composed of extracellular vesicles of 30–300 nm in size and consistent with the known morphology of exosomes (Supplementary File 1, Figure S1C). Further, exosomal size distribution was confirmed by nanoparticle tracking analysis in Nanosight (Supplementary File 1, Figure S1D). No differences were observed in the nanoparticle analysis regarding the size of miRNAs between cases and controls. Most of the detected nanoparticles were in the normal range for exosomes being ~30–300 nm in diameter.

### ExomiR Expression Profile in Human Breast Milk From Type 1 Diabetes and Healthy Control Mothers

ExomiRs were extracted and profiled using small RNA sequencing. Before quality control (QC) and filtering, on average 10 million reads per sample were obtained. The overall read distribution before and after QC is shown in Supplementary File 1 (Figure S2A). Among the filtered reads after QC, 27.6 million reads from type 1 diabetes and 34.1 million reads from control group successfully mapped to mature and precursor miRNAs from miRBase v21 (http://www.mirbase.org). These miRNA reads amounted to 10.2 and 13.2% of the total reads in type 1 diabetes and control group, respectively.

**Figure 1:**
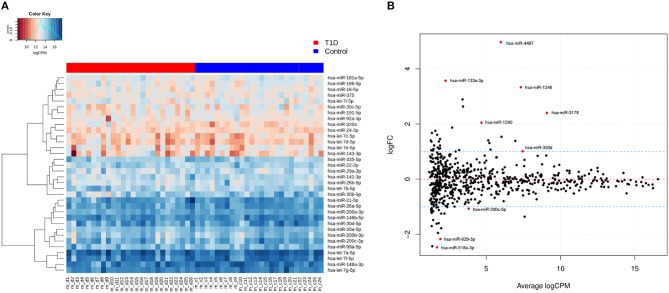
Expression profiles of known miRNAs in human breast milk. **(A)** Heatmap of expression profiles (logCPM) of top 35 highly expressed miRNAs in both type 1 diabetes and control libraries based on hierarchical clustering. **(B)** Differentially expressed miRNAs in type 1 diabetes vs. controls based on log2FC≥ abs (1) and adj. *p*-value < 0.05. A total of 9 differentially expressed miRNAs are shown in above plot. The dotted blue lines represent the log2FC cutoff.

In total, 631 unique miRNAs were found expressed in type 1 diabetes and control samples using a cutoff of CPM>1 in at least 25% of the samples (Supplementary File 1, Figure S2B). On average, 523 miRNAs were found expressed in type 1 diabetes and control libraries (Supplementary File 1, Figure S2B). The most abundant miRNAs in both groups are shown in Figure 1A. hsa-let-7a-5p, hsa-miR-148a-3p, hsa-miR-146b-5p, hsa-let-7f-5p, hsa-let-7g-5p, hsa-miR-21–5p, hsa-miR-26a-5p, and hsa-miR-30d-5p were the most highly expressed exomiRs and accounted for ~50% of the total read counts of all 631 unique miRNAs. Immune-related miRNAs such as hsa-let-7c, hsa-miR-21-5p, hsa-miR-200b-3p, hsa-miR-146a-5p, and hsa-miR-146b-5p were highly expressed based on the sequencing data (Figure 1A).

#### Differentially Expressed ExomiRs

The normalized read counts (logCPM) and fold changes for the differentially expressed miRNAs are presented in Table 2. In total, six miRNAs (hsa-miR-4497, hsa-miR-1246, hsa-miR-133a-3p, hsa-miR-3178, hsa-miR-1290, and hsa-miR-320d) were up-regulated and three miRNAs (hsa-miR-518e-3p, hsa-miR-629–3p, and hsa-miR-200c-5p) were down-regulated using the cutoff of log2FC≥ abs(0.5) and adj. *p*-value < 0.05 (Figure 1B and Table 2). The expression of these nine differentially expressed miRNAs was also tested in plasma from the same mothers but did not show comparable expression levels and no differential expression (data not shown).

**Table 2:** Differentially expressed exomiRs in type 1 diabetes vs. controls.

**miRNA**	**logFC**	**logCPM**	**LR**	***P*-value**	**FDR**
**Up-regulated miRNAs**					
hsa-miR-4497	4.97	5.98	24.91	6.02E-07	1.90E-04
hsa-miR-1246	3.33	7.33	34.08	5.28E-09	3.33E-06
hsa-miR-133a-3p	3.57	2.26	11.59	6.64E-04	0.047
hsa-miR-3178	2.40	9.08	16.53	4.78E-05	0.010
hsa-miR-1290	2.05	4.68	12.95	3.21E-04	0.036
hsa-miR-320d	1.01	7.45	11.87	5.70E-04	0.046
**Down-regulated miRNAs**					
hsa-miR-518e-3p	−2.47	1.68	11.82	5.87E-04	0.046
hsa-miR-629–3p	−2.17	1.91	12.85	3.38E-04	0.036
hsa-miR-200c-5p	−1.07	3.82	13.80	2.03E-04	0.032

*Log fold changes (logFC) and logCPM values for up-regulated and down-regulated miRNAs*.

#### Novel ExomiRs

**Figure 2:**
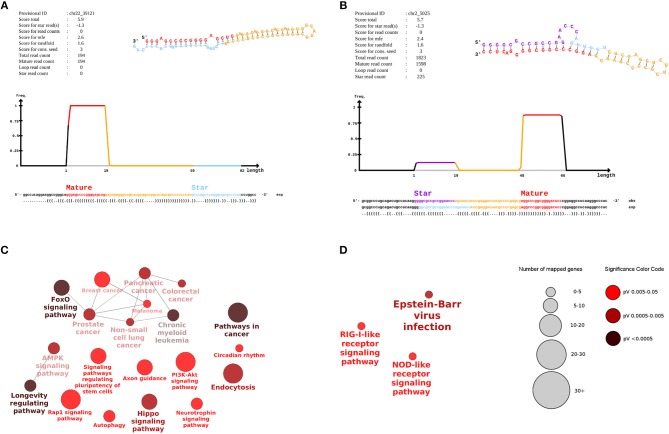
Novel miRNAs in human breast milk-derived exosomes and pathways associated with differentially expressed miRNAs. The **(A,B)** displays signature and structure of two novel miRNAs. **(A)** provisional ID: chr22_39121, located in an intron of *SUN2* **(B)** provisional ID: ch2_5025, mapped to a conserved intergenic region. The upper right figure shows the predicted RNA secondary structure of the hairpin, partitioned according to miRNA biogenesis: red, mature; yellow, loop; purple, star. The middle density plot shows the distribution of reads in the predicted precursor sequence. The sequences below indicate the positions of the mature, loop, and star strand. The positions of the star strand as expected from Drosha/Dicer processing is shown in light blue, while the star consensus positions as observed from the sequencing data is shown in purple. The **(C,D)** display a network of enriched pathways associated with targets of **(C)** upregulated and **(D)** downregulated miRNAs. The size and color of the nodes is proportional to the number of mapped genes and *p*-value significance of a given pathway.

We detected 213 potential novel miRNAs at a stringent cutoff of miRDeep2 score ≥5 and an estimated true positive probability of 78 ± 3%. In total, 208 candidate novel miRNAs remained after filtering for loci mapping to other RNA genes (Supplementary File 1, Table S1). Two highly expressed novel miRNA candidates are shown in Figure 2. The novel miRNA in Figure 2A mapped to an intron of the *SUN2* gene whereas the novel miRNA in Figure 2B mapped to a conserved region, which is not annotated. In both novel miRNAs, number of reads mapping to the predicted miRNA loop is zero. These miRNAs show the characteristic pattern of higher number of reads mapping to the mature miRNA over the star and loop sequence. Reads mapping to both mature and star miRNA strands for novel miR chr2_5025 were significantly higher in type 1 diabetes samples as compared to controls (*p*-value 2.01e-04, paired *t*-test), indicating an upregulation of this novel miRNAs in the diseased condition. The average number of reads per sample for novel miR chr2_5025 in type 1 diabetes and control samples was 13 and 3.1 respectively. Novel miR chr2_5025 was present in >80% of the samples in both conditions. However, novel miR chr22_39121 was lowly expressed and detected in only 20% of control samples and 54% of type 1 diabetes samples. The average read count for novel miR chr22_39121 was 3.1 and 2.1 in type 1 diabetes and control samples, respectively.

### Network and Pathway Analysis of exomiR Targets

We identified known target genes for the 9 differentially expressed miRNAs and performed pathway annotation and enrichment analysis. The target genes for all of the up-regulated and down-regulated miRNAs are listed in Supplementary File 2 (Table S1). The network view of both the shared as well as unique target genes of up and down-regulated miRNAs are shown as Supplementary File 1 (Figures S3, S4). The detailed pathway analysis and their associated genes are listed in Supplementary File 2 (Tables S2, S3).

The target prediction analysis identified a total of 38 target genes for the most highly up-regulated miRNA hsa-miR-4497 (Supplementary File 2, Table S1). Among its target genes, *CXCR5, BMP8A, CCNF*, and *VEGFA* were found particularly interesting. *VEGFA* (Vascular Endothelial Growth Factor A) specifically targets both hsa-miR-4497 and hsa-miR-133a-3p and has been shown to be involved in angiogenesis, PI3k/Akt signaling and VEGF signaling. *BMP8A* (Bone morphogenetic protein 8A), another target for miR-4497 is involved in TGF-beta signaling pathway. *CCNF* (Cyclin F), which is an important regulator of cell cycle processes is also targeted by miR-4497. *CCNF* is associated with various pathways including DNA damage, Class I MHC mediated antigen processing and presentation and innate immune system.

The target genes for the 6 up-regulated miRNAs were associated with 20 enriched pathways including key signaling and endocytosis pathways such as AMPK, FoxO, Rap1 signaling, PI3K-Akt signaling (Figure 2C). FoxO signaling and longevity-regulating pathways were the most significant pathways associated with the up-regulated miRNA targets (*p* < 0.0005). For the 3 down-regulated miRNAs, their target genes were associated with three enriched pathways including NOD-like receptor, RIG-1 like receptor and Epstein-Barr virus infection (Figure 2D).

### hsa-miR-4497 and hsa-miR-3178 Increases TNFα Production in Human Macrophages

**Figure 3:**
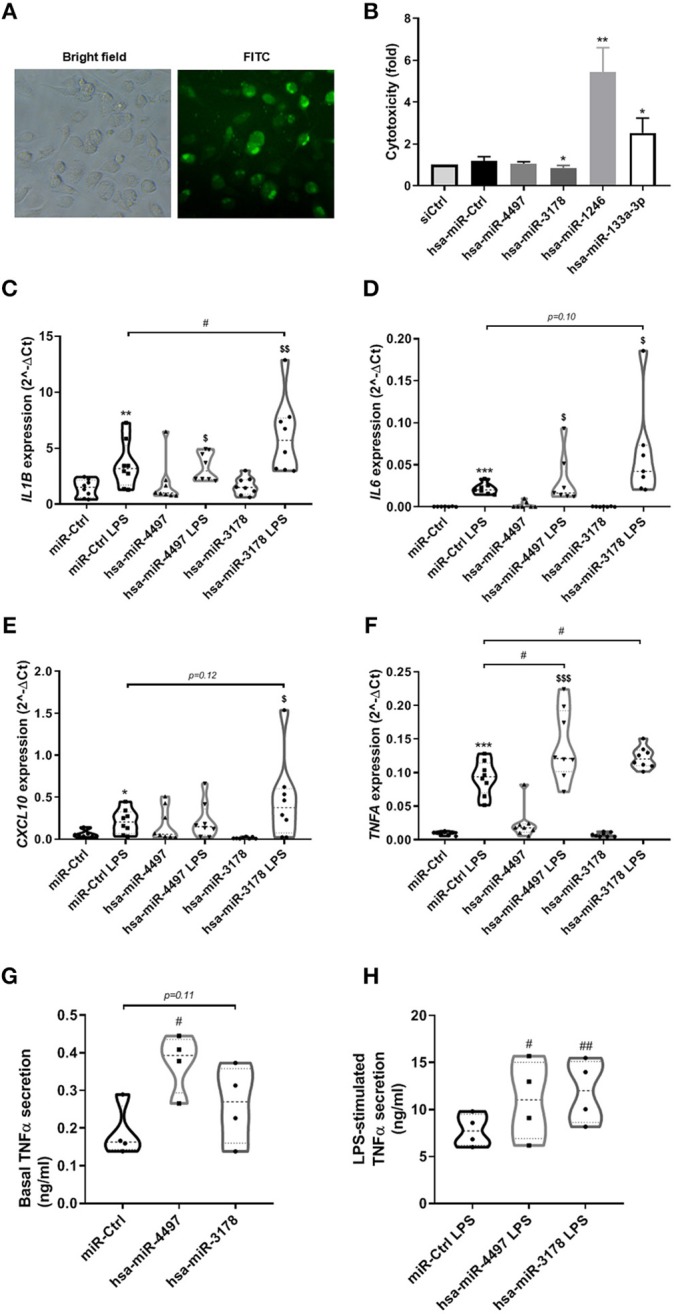
hsa-miR-4497 and hsa-miR-3178 augments LPS-induced *TNF*α expression and secretion. Fluorescence microscopy of human THP-1 cells transfected with siGLO. **(B)** THP-1 cells transfected with negative control miRNA (miR-Ctrl) or hsa-miR-4497, hsa-miR-1246, hsa-miR-133a-3p, and hsa-miR-3178 were subject to cytotoxicity assay 2 days post transfection to determine cell viability. Data are means ± SEM of *n* = 5. **(C–F)** Gene expression in THP-1 cells transfected as in **(B)** and exposed to 5 ng/ml LPS for 3 h was measured by realtime PCR and normalized to that of *ACTB*. Data are means ± SEM of *n* = 8. *, **, ***p < 0.05, 0.01, 0.001, respectively, vs. miR-Ctrl. ^*$*^, ^*$$*^*p* < 0.05, 0.01, respectively, vs. hsa-miR-4497 and hsa-miR-3178. ^#^*p* < 0.05, One-way ANOVA with multiple comparisons **(A–F)**. Basal **(G)** and LPS-induced **(H)** TNFα secretion to the culture medium during 3 h from human THP-1 cells transfected with negative control miRNA (miR-Ctrl) or hsa-miR-4497 and hsa-miR-3178. Data are means ± SEM of *n* = 4. ^#^*p* ≤ 0.05, paired *t*-test **(G,H)**.

The most highly upregulated exomiRs in the milk of mothers with type 1 diabetes were further explored for potential effect on immune cell activity. Human monocytic THP-1 cells were differentiated into macrophages and transfected with hsa-miR-4497, hsa-miR-1246, hsa-miR-133a-3p, and hsa-miR-3178 mimics. Control transfection experiments using a fluorescent siRNA molecule confirmed high transfection efficiency (Figure 3A). Two of the miRNAs (hsa-miR-1246 and hsa-miR-133a-3p 2) were found to be cytotoxic to the cells as measured by cytotoxicity assay and hence were not investigated further (Figure 3B). Examination of lipopolysaccharide (LPS)-induced expression of classical pro-inflammatory genes (*IL1B, IL6, CXCL10*, and *TNF*α) revealed that hsa-miR-4497 augmented LPS-stimulated TNFα expression (Figures 3C–F). In line with this, hsa-miR-4497 transfected cells secreted more TNFα to the culture medium compared to control-transfected cells both under basal and LPS conditions (Figures 3G,H). Transfection with hsa-miR-3178 also increased expression of TNFα and *IL1B* at the transcriptional level and significantly increased secreted TNFα at LPS induced condition (Figures 3C,F,H).

In human epithelial CaCo-2 cells, we also observed high transfection rate with fluorescent siRNA (Supplementary File 1, Figure S5A). None of the miRNA mimics were cytotoxic to the CaCo-2 cells (Supplementary File 1, Figure S5B). Transfection with hsa-miR-4497, hsa-miR-1246, hsa-miR-133a-3p, and hsa-miR-3178 mimics showed minor or very little effect on the expression of *IL1B, IL6, TNF*α, *and CXCL10* compared to negative control miR-transfected cells (*n* = 3) (Supplementary File1, Figure S5).

### Effect of miRNA Expression on Clinical Parameters

Overall weak positive correlations were observed between the expression levels of up-regulated miRNAs and HbA1c, whereas the down-regulated miRNAs showed weak negative correlations with HbA1c. There was no difference in correlation between the two groups (Supplementary File 1, Figures S6, S7). We also tested the additive effects of maternal age and BMI on HbA1c in the regression analysis, however no significant effects were observed (data not shown).

## Discussion

It is long known that human breast milk provides optimal nutrition and contributes to the shaping of the mucosal immunity in infants; however, the molecular mechanisms that enable the transfer of immunity are incompletely understood. Given the emerging roles of miRNAs as crucial mediators of immune cognition (20–23), we sought to investigate both their presence and function in human breast milk from mothers with type 1 diabetes and healthy controls. To our knowledge, this is the first study to investigate the differences in expression of human breast milk miRNAs from type 1 diabetes mothers compared to healthy controls.

A total of 631 unique miRNAs were found expressed in type 1 diabetes and control samples supporting the high presence of miRNAs in human breast milk. Interestingly, hsa-miR-7a-5p and hsa-miR-148a-3p alone accounted for ~18% of the total read counts of all miRNAs detected in human breast milk. This is consistent with previous reports, where hsa-miR-148a-3p was found to be present in high concentrations in bovine milk and was identified as a potential biomarker for the quality control of raw milk and other milk-related products (17). In total, eight highly expressed exomiRs (hsa-let-7a-5p, hsa-miR-148a-3p, hsa-miR-146b-5p, hsa-let-7f-5p, hsa-let-7g-5p, hsa-miR-21–5p, hsa-miR-26a-5p, and hsa-miR-30d-5p) accounted for ~50% of the total read counts. All of these highly expressed miRNAs are associated with inflammatory and immune responses as shown by other studies (20–22).

Nine miRNAs were differentially expressed between type 1 diabetes and control samples. These include hsa-miR-4497, hsa-miR-1246, hsa-miR-133a-3p, hsa-miR-3178, hsa-miR-1290, hsa-miR-320d, hsa-miR-518e-3p, hsa-miR-629–3p, and hsa-miR-200c-5p. Majority of their target genes are involved in cell cycle and immune-response pathways including FoxO signaling, longevity regulating pathway, autophagy, endocytosis, AMPK signaling, Rap1 signaling, PI3K-Akt signaling. Genes involved in FoxO signaling included TGF-β signaling pathway-associated genes of SMAD family member 4 (*SMAD4*), transforming growth factor beta 2 (*TGFB2*), *TGFBR1* and *TGFBR2*. The TGF-β mediated pathways are central regulators of cell proliferation and survival, migration, and differentiation and angiogenesis (24). Importantly, inhibition of TGF- β signaling has been shown to promote β-cell replication in adult mice and grafted human islets (24). The target genes involved in both FoxO signaling and longevity-regulating pathways included *AKT3, FOXO3, IGF1, IGF1R, INSR, KRAS, PIK3R1, PIK3R2*, and *SIRT1*. In the immune system, autophagy functions range from the elimination of infectious agents and the modulation of the inflammatory response, to the selection of antigens for presentation and the regulation of T cell homeostasis and activation (25–27). It is also involved in immune receptor-mediated vacuolar cell death as well as programmed cell death (19). Various studies have shown that autophagy is responsive to alterations in PI3K signaling. The target genes of the up-regulated miRNAs that are associated with PI3K-Akt signaling included *PIK3R2, PIK3R1, GSK3B, VEGFA, CCND2, GNB4, GRB2, PTEN, AKT3, FOXO3, CASP9, INSR*, and *PDPK1*.

Previous studies reported a link between PI3K/Akt signaling and production of cytokines including TNF-α and IL-6 in LPS induced macrophages (28–30). Tessaro et al. showed that insulin treatment enhances LPS-induced cytokine secretion in bone marrow-derived macrophages from diabetic mice via PI3K and ERK1/2 (28). In non-diabetic alveolar macrophages, insulin inhibits LPS-induced p38 and ERK 1/2 MAPK, PKC, and Akt phosphorylation, resulting in reduced TNF-α levels (31). These studies suggest a positive regulation of TNF-α by PI3K/Akt. In our study, we found that, hsa-miR-4497 and hsa-miR-3178 mimics enhanced TNF-α secretion in differentiated THP1 cells compared to control-transfected cells both under basal and LPS induced conditions. However, similar effects on the gene expression of studied inflammatory markers were not observed in human epithelial Caco-2 cells transfected with the miR-mimics. Furthermore, there was no significant effect of the miRNA-mimics on TNF-α secretion in CaCo-2 cells (data not shown) highlighting that the cellular effects of these miRNAs are cell type specific.

Several miRNAs in natural regulatory CD4^+^ FOXP3^+^ T cells (nTreg) have been found as markers of disease risk and T cell dysfunction in high-risk type 1 diabetes individuals (32). Noteworthy, one study found significant up-regulation of miR-200c in *in vivo* activated nTreg cells compared to naive nTreg in pre-type 1 diabetes individuals (33). In the same study, miR-629 was also up-regulated in aTregs in pre-type 1 diabetes individuals compared to healthy controls. Contrary to this, we observed significantly down-regulation of these miRNAs in milk from mothers with type 1 diabetes, which may suggest a potential beneficiary role of these miRNAs to the infants immune cells. However, further validation is required to determine the contribution of these miRNAs in T-cell function/dysfunction. Interestingly, when tested in plasma samples from the same mothers, none of these nine miRNAs showed differential expression, indicating that the breastmilk miRNA profile is not reflected in the blood. This is in agreement with a recent study (34).

We also identified 208 novel miRNAs in this study. All of the predicted novel miRNAs shared the same seed sequence with known miRNAs from other species, which increases the likelihood that these are true miRNAs. Most of these novel miRNAs are missed by traditional analysis due to low expression levels and because they are located within unannotated regions of the genome—or they might be breastmilk specific. Recent studies have identified novel tissue-specific miRNAs with a proportional distribution across the genome similar to already known miRBase-cataloged miRNAs (35, 36). We found that two of the highest expressed novel miRNAs showed significantly higher levels in breast milk of mothers with type 1 diabetes. Additional experimental work will be required to functionally validate the highly abundant novel miRNA candidates identified in this study.

Human breast milk derived exosomes are capable of influencing the infant's intestinal immune response as well as the local immune response to bacterial challenge (15, 37). Näslund et al. demonstrated that pre-exposure to human breast milk derived exosomes and not plasma-derived exosomes, reduced productive HIV-1 infection of monocyte-derived dendritic cells and subsequent viral transfer to CD4^+^ T cells (38). Recent studies have also proved that breast milk derived exosomes can enter human intestinal crypt-like cells suggesting that their cargo, mainly exomiRs, and mRNAs, might be able to alter the protein expression at the neonatal mucosal surface (4, 15). Noteworthy, exogenous miRNAs have been experimentally proven to regulate gene expression in mammalian cells (37, 39). These miRNAs are transferred via food intake, and it is likely that the same transfer of miRNA to the breastfed infant occurs, particularly since the neonatal stomach is less acidic and the gut highly leaky early in life (40). Manca et al. studied the bioavailability and distribution of miRNAs in bovine, porcine and murine milk exosomes and observed unique distribution profiles (41). Milk exosomes accumulated in intestinal mucosa, spleen, liver, heart, and brain following suckling oral gavage and intravenous administration in mice and pigs (41). Synthetic miRNAs transfected into bovine milk exosomes administered to mice also demonstrated accumulation in intestinal mucosa, spleen, liver, heart, and brain (41). Another study found uptake of milk-derived miRNA by mammalian cells and a therapeutic function in ameliorating experimental arthritis in mice (42). It can also be hypothesized that breastmilk miRNAs may act directly on the infants microbiome as breastfeeding is known to be associated with changes in the infant gut microbiota (43). Thus, the demonstration of specific miRNA profiles in breast milk further supports its potential role in influencing infant development and health.

The network and pathway analyses of miRNA targets in our study showed enrichment of key signaling and endocytosis pathways that also might have an influence on the infant's immune system. To seek proof-of-concept for this hypothesis, we examined the effect of hsa-miR-4497 (highest differentially expressed miR) on human monocytic THP-1 cells. Lipopolysaccharide-induced expression of classical pro-inflammatory genes revealed that hsa-miR-4497 augmented LPS-stimulated TNFα expression. In line with this, hsa-miR-4497-transfected cells secreted more TNFα to the culture medium compared to control-transfected cells both under basal and LPS conditions. The consequence(s) of this is not known but support that breast milk-derived miRNAs may have immune-regulatory effects in the newborn. In a recent study, Nojehdehl et al. demonstrated that exosomes derived from adipose tissue-derived mesenchymal stem cells (AD-MSCs) have immunomodulatory effects of T-cell inflammatory response and reduction of clinical symptoms in streptozotocin-induced of the type-1 diabetes model (44). For future studies, it might be promising to add breast milk exosomes to streptozotocin-induced type 1 diabetes models to observe potential variations in beta-cell survival or apoptosis.

Half of the mothers with type 1 diabetes used artificial infant formulae as supplement to breastfeeding compared to 3 out of 26 control mothers. However, it has been firmly established that bovine milk-based and soy-based formulae contain very few human mature miRNAs (34), so this is unlikely to influence the observations in this study. Furthermore, there were no clear correlations of miRNA levels to clinical data (BMI, HbA1c).

The present data demonstrates that there are differences in levels of miRNAs in breast milk from mothers with type 1 diabetes and healthy mothers. If this has any effect on the risk of type 1 diabetes in the infants is currently not known. Thus, these results do not change the fact that women, independent of diabetes status, should breastfeed according tobreak current recommendation.

## Data Availability Statement

The datasets generated for this study can be found in ArrayExpress, E-MTAB-7336.

## Ethics Statement

All women involved in the study gave their signed informed consent to participate. The study was approved by the Ethical Committee for the Capital Region, Denmark (H-4–2013-008).

## Author Contributions

LN, HM, and FP conceived, designed, and managed the study. AM, SK, RY, and FP prepared and analyzed samples. AM, SK, and MR did further sample characterization. LN, EM, PD, and JSv recruited participants and assembled phenotypic data. HM and FP secured resources and facilities for the research. AM, SK, JSt, and FP performed the analysis, interpreted data and wrote the manuscript with input from all authors. All authors read and approved the final manuscript before submission. FP had full access to all the data in the study and takes full responsibility for the integrity of the data and the accuracy of the data analysis.

### Conflict of Interest

The authors declare that the research was conducted in the absence of any commercial or financial relationships that could be construed as a potential conflict of interest.
