# Supplementary material for: Breast Milk-Derived Extracellular Vesicles Enriched in Exosomes From Mothers With Type 1 Diabetes Contain Aberrant Levels of microRNAs
Source: Front Immunol. 2019 Oct 25;10:2543. doi: 10.3389/fimmu.2019.02543 (PMC6823203; doi:10.3389/fimmu.2019.02543)
Supplement: Supplementary file 1 [file Data_Sheet_1.docx]

**Supplementary File 1**

**Part1: Additional Methods**

**A. Exosome characterization**

Purified exosomes were further analyzed for the presence of canonical exosome surface markers: CD81, CD63 and HSP70 by Western blot analysis. Briefly, exosomes in 1X PBS were solubilized with ice-cold RIPA Lysis and Extraction Buffer (#89900, Thermo Scientific, Waltham, MA, USA) supplemented with protease inhibitor solution cOmplete, EDTA-free (# 11 837 580 001, Roche Applied Science, Mannheim, Germany). Total protein concentration of exosome lysate was quantified using Quick Start™ Bradford Protein Assay (#5000201, BioRad, Hercules, CA, USA) according to the manufacturer's instructions. Onto a 10% SDS-polyacrylamide gel, three lanes were loaded each with 25μg of exosomal protein and next to each sample lane a Precision Plus Protein™ Kaleidoscope™ (#1610375, BioRad, Hercules, CA, USA) prestained protein ladder was loaded and resolved by electrophoresis. Exosomal proteins were transferred to Immobilon-P PVDF membrane (#INCP00010, Millipore Sigma, Burlington, MA, USA). For immunoblotting, the membrane was cut horizontally into three equal sections along the protein marker lanes. Each section were probed separately with a primary antibody. Primary antibodies used were; rabbit polyclonal anti-CD81 (1:1000 dilution), CD63 (1:500 dilution) (sc-9158 and sc-15363, Santa Cruz Biotechnology, Dallas, TX, USA), and HSP70 (1:1000 dilution) (4872, Cell Signaling). HRP-conjugated anti-rabbit IgG secondary antibody (#7074, Cell Signaling Technology, Danvers, MA, USA) was used to detect immune complexes were by chemiluminescence using Amersham ECL Western Blotting Detection Kit (#RPN2108, Chicago, IL, USA). The blot images were digitally captured using a FUJI LAS4000 imaging system (Fujifilm, Tokyo, Japan).

*Transmission electron microscopy*

Identification of exosomes was confirmed by the transmission electron microcopy (TEM). Briefly, the sample containing resuspended exosomes in PBS were stained for 1 min with 1% uranyl acetate. Excess fluid was removed with a piece of Whatman filter paper. All transmission electron micrographs were obtained using CM 100(a) electron microscopy at 100 kv.

*Nanosight based nanoparticle tracking analysis*

NanoSight based exosome characterization was performed based using a Nanosight LM20 (Nanosight, Amesbury, UK) on nanoparticle tracking analysis (NTA 3·0). Briefly, 5 µl of resuspended exosome sample diluted 500X with 1X PBS buffer was used for the NTA analysis. 3 random samples were measured 5 consecutive times at 3 independent days.

**B. Small RNA sequencing**

The small RNAseq was performed at New York Genome Center (NYGC) using TruSeq Small RNA Library Preparation Kit (#RS-200-0048; Illumina, San Diego, CA, USA).Briefly, raw reads were trimmed for adapters and first and last four bases were clipped to remove low quality ends using Trimmomatic (1). Further, reads were filtered based on average quality cut-off of Q30 and a minimum length threshold of 10 bases. Filtered reads were collapsed so that each sequence is only represented once. Reads were then mapped to human genome (GRCh38) and human mature and precursor miRNAs based on miRBase v·21 using Seqbuster-miRaligner (2, 3). The reads were aligned and mapped to mature miRNAs using default parameters in miRaligner (i.e. one mismatch, 3 nt for the 3′ or 5′ trimming variants, 3 nt for the 3′-end addition variants). The number of reads mapping to each mature miRNA was counted and normalized using reads per million (CPM) in EdgeR package in R (4). The miRNAs were filtered using a cutoff of CPM >1 in at least 10 samples. Differential expression analysis was carried out in EdgeR with a cutoff of log2FC≥ abs(0·5) and adjusted p-value <0·05. In total, 270 and 258 million raw reads were obtained for T1D and control samples, respectively. After trimming and filtering low quality reads, 222 and 208 million high quality reads remained for further analysis in T1D and control group, respectively.

**C. Prediction of novel exomiRs in human breast milk**

The filtered reads from T1D and control samples were further analyzed with miRDeep2 (5), to detect novel miRNAs. Briefly, the filtered collapsed reads were aligned to human genome (GRCh38, Gencode v25) using miRDeep2. Known miRNAs from human and four other species were used as input for more reliable prediction of novel miRNAs with conserved seeds in other species. Pan troglodytes (ptr), Pan paniscus (ppa), Gorilla gorilla (ggo) and Pongo pygmaeus (ppy) were designated as related species for the purpose of input miRNAs.

**D. miRNA target prediction and pathway analysis**

#### The CytoScape plugin CyTargetLinker v 3·01 (6) was used for identifying gene targets for each miRNA and to create miRNA-target interaction (MTI) networks. Regulatory interaction networks (RegIN) were created within CyTargetLinker in Cytoscape using Homo sapiens MTIs from an experimentally validated miR-target resource miRTarBase v6·1 (7) which includes 410,602 MTIs, and a predicted miR-target resource TargetScan v6·2 (8) which includes 511,040 MTIs. Targets supported by any of these two resources were retained and visualized in networks. Pathway annotation network analysis of miRNA targets was perfomed using CytoScape plugin ClueGO (9).

**References**

1. Bolger AM, Lohse M, Usadel B. Trimmomatic: a flexible trimmer for Illumina sequence data. *Bioinformatics* (2014) **30**: 2114–20.
2. Kozomara A, Griffiths-Jones S. miRBase: annotating high confidence microRNAs using deep sequencing data. *Nucleic Acids Res* (2014) **42**: D68-73.
3. Pantano L, Estivill X, Martí E. SeqBuster, a bioinformatic tool for the processing and analysis of small RNAs datasets, reveals ubiquitous miRNA modifications in human embryonic cells. *Nucleic Acids Res* (2010) **38**: e34.
4. McCarthy DJ, Chen Y, Smyth GK. Differential expression analysis of multifactor RNA-Seq experiments with respect to biological variation. *Nucleic Acids Res* (2012) **40**: 4288–97.
5. Friedländer MR, Mackowiak SD, Li N, Chen W, Rajewsky N. miRDeep2 accurately identifies known and hundreds of novel microRNA genes in seven animal clades. *Nucleic Acids Res* (2012) **40**: 37–52.
6. Kutmon M, Kelder T, Mandaviya P, Evelo CTA, Coort SL. CyTargetLinker: a cytoscape app to integrate regulatory interactions in network analysis. *PLoS ONE* (2013) **8**: e82160.
7. Chou C-H, Chang N-W, Shrestha S, *et al.* miRTarBase 2016: updates to the experimentally validated miRNA-target interactions database. *Nucleic Acids Res* (2016) **44**: D239-247.
8. Agarwal V, Bell GW, Nam J-W, Bartel DP. Predicting effective microRNA target sites in mammalian mRNAs. *Elife* (2015) **4**. DOI:10.7554/eLife.05005.
9. Bindea G, Mlecnik B, Hackl H, *et al.* ClueGO: a Cytoscape plug-in to decipher functionally grouped gene ontology and pathway annotation networks. *Bioinformatics* (2009) **25**: 1091–3.

**Part 2: Additional Results**

**Table S1: Novel miRNAs detected by miRDeep2**

| **provisional id** | **precursor coordinate** | **miRDeep2 score** | **estimated probability that the miRNA candidate is a true positive** | **total read count** | **mature read count** | **loop read count** | **star read count** | **significant randfold p-value** | **example miRBase miRNA with the same seed** |
| --- | --- | --- | --- | --- | --- | --- | --- | --- | --- |
| chr22_39121 | chr22:38750750..38750831:- | 5.9 | 78 +/- 3% | 194 | 194 | 0 | 0 | yes | gga-miR-6561-3p |
| chr4_11081 | chr4:185017142..185017186:- | 5.9 | 78 +/- 3% | 127 | 127 | 0 | 0 | yes | dps-miR-2532 |
| chr12_26576 | chr12:8018707..8018776:- | 5.9 | 78 +/- 3% | 108 | 86 | 3 | 19 | yes | eca-miR-675 |
| chr6_15061 | chr6:41637102..41637158:- | 5.9 | 78 +/- 3% | 73 | 73 | 0 | 0 | yes | esi-miR3459-5p |
| chr3_9059 | chr3:184315102..184315148:- | 5.9 | 78 +/- 3% | 409 | 409 | 0 | 0 | yes | mmu-miR-5126 |
| chr11_23783 | chr11:32434896..32434956:+ | 5.9 | 78 +/- 3% | 129 | 129 | 0 | 0 | yes | mmu-miR-3960 |
| chr17_33874 | chr17:44558239..44558288:- | 5.8 | 78 +/- 3% | 462 | 462 | 0 | 0 | yes | mml-miR-939 |
| chr16_32568 | chr16:88737503..88737585:- | 5.8 | 78 +/- 3% | 494 | 494 | 0 | 0 | yes | bta-miR-2327 |
| chr7_16859 | chr7:1665185..1665251:- | 5.8 | 78 +/- 3% | 176 | 176 | 0 | 0 | yes | dre-miR-139-3p |
| chr19_35260 | chr19:1790018..1790079:+ | 5.8 | 78 +/- 3% | 171 | 171 | 0 | 0 | yes | mghv-miR-M1-2-5p |
| chr1_1928 | chr1:9652447..9652515:- | 5.8 | 78 +/- 3% | 70 | 70 | 0 | 0 | yes | blv-miR-B4-5p |
| chr8_18725 | chr8:132481096..132481169:+ | 5.8 | 78 +/- 3% | 77 | 77 | 0 | 0 | yes | blv-miR-B4-5p |
| chr22_39276 | chr22:49912080..49912150:- | 5.8 | 78 +/- 3% | 4307 | 4307 | 0 | 0 | yes | efu-miR-9205 |
| chr19_35518 | chr19:18711586..18711656:+ | 5.8 | 78 +/- 3% | 23 | 23 | 0 | 0 | yes | ptc-miR319i |
| chr17_33009 | chr17:42458785..42458853:+ | 5.8 | 78 +/- 3% | 377 | 377 | 0 | 0 | yes | mmu-miR-128-1-5p |
| chr2_5025 | chr2:230848084..230848149:+ | 5.7 | 78 +/- 3% | 1823 | 1598 | 0 | 225 | yes | ppy-miR-1538 |
| chr14_28941 | chr14:75176170..75176223:+ | 5.7 | 78 +/- 3% | 61 | 60 | 0 | 1 | yes | mmu-miR-3104-5p |
| chr5_12115 | chr5:168165854..168165921:+ | 5.7 | 78 +/- 3% | 12 | 12 | 0 | 0 | yes | cel-lin-4-3p |
| chr3_8322 | chr3:49663513..49663590:- | 5.7 | 78 +/- 3% | 12 | 12 | 0 | 0 | yes | mmu-miR-5129-5p |
| chr16_31143 | chr16:1089765..1089844:+ | 5.7 | 78 +/- 3% | 265 | 265 | 0 | 0 | yes | cin-miR-4002-3p |
| chr1_1432 | chr1:203034715..203034766:+ | 5.7 | 78 +/- 3% | 17 | 17 | 0 | 0 | yes | mmu-miR-3475-3p |
| chr8_19821 | chr8:144509841..144509904:- | 5.7 | 78 +/- 3% | 3840 | 3840 | 0 | 0 | yes | ppt-miR171a |
| chr19_36143 | chr19:14057366..14057410:- | 5.7 | 78 +/- 3% | 269 | 269 | 0 | 0 | yes | mmu-miR-5110 |
| chr2_5115 | chr2:238947449..238947491:+ | 5.7 | 78 +/- 3% | 154 | 154 | 0 | 0 | yes | blv-miR-B4-5p |
| chr10_22158 | chr10:89197656..89197717:+ | 5.7 | 78 +/- 3% | 123 | 120 | 0 | 3 | yes | ppt-miR2080 |
| chr4_9144 | chr4:53552..53599:+ | 5.7 | 78 +/- 3% | 60 | 60 | 0 | 0 | yes | mmu-miR-3960 |
| chr7_17703 | chr7:140176223..140176258:- | 5.7 | 78 +/- 3% | 117 | 117 | 0 | 0 | yes | bta-miR-2890 |
| chr3_9121 | chr3:194297112..194297196:- | 5.7 | 78 +/- 3% | 401 | 401 | 0 | 0 | yes | mmu-miR-3960 |
| chr20_37177 | chr20:842285..842356:- | 5.7 | 78 +/- 3% | 43 | 43 | 0 | 0 | yes | dme-miR-310-5p |
| chr14_29088 | chr14:95432417..95432465:+ | 5.6 | 78 +/- 3% | 13 | 13 | 0 | 0 | yes | smo-miR1090 |
| chr1_3585 | chr1:247911263..247911351:- | 5.6 | 78 +/- 3% | 181 | 181 | 0 | 0 | yes | bmo-miR-14-5p |
| chr4_9154 | chr4:386375..386426:+ | 5.6 | 78 +/- 3% | 60 | 60 | 0 | 0 | yes | mmu-miR-3960 |
| chr19_36340 | chr19:35780869..35780943:- | 5.6 | 78 +/- 3% | 39 | 39 | 0 | 0 | yes | ppt-miR1057 |
| chr6_14941 | chr6:31016925..31016978:- | 5.6 | 78 +/- 3% | 127 | 127 | 0 | 0 | yes | pab-miR3710 |
| chr17_33010 | chr17:42458833..42458916:+ | 5.6 | 78 +/- 3% | 378 | 377 | 1 | 0 | yes | mmu-miR-128-1-5p |
| chr11_23580 | chr11:2269166..2269209:+ | 5.6 | 78 +/- 3% | 164 | 164 | 0 | 0 | yes | mmu-miR-1893 |
| chr1_3229 | chr1:203795907..203795958:- | 5.6 | 78 +/- 3% | 30 | 30 | 0 | 0 | yes | rlcv-miR-rL1-22-5p |
| chr9_20668 | chr9:135501332..135501412:+ | 5.6 | 78 +/- 3% | 103 | 103 | 0 | 0 | yes | bta-miR-7860 |
| chr4_9213 | chr4:3450081..3450121:+ | 5.6 | 78 +/- 3% | 122 | 122 | 0 | 0 | yes | zma-miR399d-5p |
| chr20_37717 | chr20:63068281..63068327:- | 5.6 | 78 +/- 3% | 142 | 142 | 0 | 0 | yes | mmu-miR-3064-5p |
| chr17_32997 | chr17:41328415..41328481:+ | 5.6 | 78 +/- 3% | 45 | 45 | 0 | 0 | yes | gma-miR390e |
| chrX_40103 | chrX:149963331..149963398:+ | 5.6 | 78 +/- 3% | 669 | 669 | 0 | 0 | yes | bra-miR9563b-5p |
| chr8_19774 | chr8:142534212..142534253:- | 5.6 | 78 +/- 3% | 758 | 758 | 0 | 0 | yes | mmu-miR-7004-3p |
| chr2_3772 | chr2:24852862..24852903:+ | 5.5 | 78 +/- 3% | 68 | 68 | 0 | 0 | yes | zma-miR399d-5p |
| chr11_24427 | chr11:118623456..118623533:+ | 5.5 | 78 +/- 3% | 474 | 474 | 0 | 0 | yes | mml-miR-636 |
| chr8_19152 | chr8:42274773..42274859:- | 5.5 | 78 +/- 3% | 77 | 64 | 13 | 0 | yes | mdo-miR-7346-5p |
| chr10_23504 | chr10:131140846..131140919:- | 5.5 | 78 +/- 3% | 36 | 36 | 0 | 0 | yes | dme-miR-2495-3p |
| chr11_23970 | chr11:62925446..62925486:+ | 5.5 | 78 +/- 3% | 245 | 245 | 0 | 0 | yes | cte-miR-2689 |
| chr16_32331 | chr16:57756602..57756674:- | 5.5 | 78 +/- 3% | 76 | 72 | 4 | 0 | yes | oan-miR-1356 |
| chr18_34371 | chr18:12008928..12008999:+ | 5.5 | 78 +/- 3% | 72 | 72 | 0 | 0 | yes | cre-miR1171 |
| chr10_22432 | chr10:122469598..122469663:+ | 5.5 | 78 +/- 3% | 128 | 128 | 0 | 0 | yes | gga-miR-1813 |
| chr7_15807 | chr7:1880869..1880928:+ | 5.5 | 78 +/- 3% | 120 | 120 | 0 | 0 | yes | sme-miR-1175-5p |
| chr5_13209 | chr5:159208181..159208241:- | 5.5 | 78 +/- 3% | 106 | 100 | 6 | 0 | yes | mmu-miR-3960 |
| chr1_964 | chr1:119735890..119735946:+ | 5.5 | 78 +/- 3% | 9512 | 9512 | 0 | 0 | yes | mdo-miR-7365-3p |
| chr22_38798 | chr22:48605930..48605982:+ | 5.5 | 78 +/- 3% | 462 | 462 | 0 | 0 | yes | rmi-miR-5326 |
| chr19_36534 | chr19:51065600..51065665:- | 5.5 | 78 +/- 3% | 72 | 72 | 0 | 0 | yes | gga-miR-1607 |
| chr8_19769 | chr8:142146255..142146332:- | 5.5 | 78 +/- 3% | 79 | 79 | 0 | 0 | yes | bta-miR-2882 |
| chr12_27344 | chr12:128664074..128664116:- | 5.5 | 78 +/- 3% | 753 | 753 | 0 | 0 | yes | cel-miR-37-5p |
| chrX_40577 | chrX:77703550..77703620:- | 5.5 | 78 +/- 3% | 44 | 44 | 0 | 0 | yes | sme-miR-281-5p |
| chr3_7564 | chr3:128362168..128362254:+ | 5.5 | 78 +/- 3% | 2899 | 2899 | 0 | 0 | yes | osa-miR1846d-5p |
| chr3_8236 | chr3:42698252..42698291:- | 5.5 | 78 +/- 3% | 71 | 71 | 0 | 0 | yes | dre-miR-19d-5p |
| chr13_27548 | chr13:42048079..42048125:+ | 5.5 | 78 +/- 3% | 2960 | 2960 | 0 | 0 | yes | bta-miR-2894 |
| chr17_32624 | chr17:2399873..2399922:+ | 5.5 | 78 +/- 3% | 110 | 110 | 0 | 0 | yes | mghv-miR-M1-2-5p |
| chr17_33568 | chr17:8131852..8131926:- | 5.5 | 78 +/- 3% | 87 | 77 | 10 | 0 | yes | vvi-miR171h |
| chr3_7617 | chr3:135191902..135191953:+ | 5.4 | 78 +/- 3% | 19 | 19 | 0 | 0 | yes | gga-miR-1784-5p |
| chr4_10149 | chr4:183619490..183619541:+ | 5.4 | 78 +/- 3% | 14 | 14 | 0 | 0 | yes | rrv-miR-rR1-7-5p |
| chr14_29496 | chr14:60643213..60643281:- | 5.4 | 78 +/- 3% | 322 | 322 | 0 | 0 | yes | mmu-miR-92b-5p |
| chr8_18161 | chr8:41343258..41343304:+ | 5.4 | 78 +/- 3% | 18354 | 18354 | 0 | 0 | yes | ame-miR-3718b |
| chr7_15808 | chr7:1880910..1880954:+ | 5.4 | 78 +/- 3% | 120 | 120 | 0 | 0 | yes | sme-miR-1175-5p |
| chr17_33321 | chr17:73878002..73878065:+ | 5.4 | 78 +/- 3% | 431 | 431 | 0 | 0 | yes | mmu-miR-92b-5p |
| chr18_34599 | chr18:57186213..57186251:+ | 5.4 | 78 +/- 3% | 4002 | 3999 | 0 | 3 | yes | bta-miR-2894 |
| chr14_29852 | chr14:104592843..104592930:- | 5.4 | 78 +/- 3% | 486 | 486 | 0 | 0 | yes | mml-miR-636 |
| chr3_8787 | chr3:133862331..133862378:- | 5.4 | 78 +/- 3% | 199 | 199 | 0 | 0 | yes | mml-miR-636 |
| chr19_36283 | chr19:29105401..29105472:- | 5.4 | 78 +/- 3% | 278 | 278 | 0 | 0 | yes | str-miR-8369-5p |
| chr10_21793 | chr10:30786339..30786379:+ | 5.4 | 78 +/- 3% | 1043 | 1043 | 0 | 0 | yes | mml-miR-134-3p |
| chr2_5105 | chr2:238448088..238448177:+ | 5.4 | 78 +/- 3% | 139 | 134 | 5 | 0 | yes | gga-miR-3539 |
| chr19_35498 | chr19:17823939..17823982:+ | 5.4 | 78 +/- 3% | 12 | 12 | 0 | 0 | yes | ptr-miR-646 |
| chr11_23996 | chr11:64626675..64626711:+ | 5.4 | 78 +/- 3% | 1432 | 1432 | 0 | 0 | yes | gga-miR-1687-5p |
| chr10_23094 | chr10:86666882..86666952:- | 5.4 | 78 +/- 3% | 12 | 12 | 0 | 0 | yes | ath-miR164a |
| chr4_10157 | chr4:184888856..184888922:+ | 5.4 | 78 +/- 3% | 69 | 69 | 0 | 0 | yes | prd-miR-7936-3p |
| chr6_15769 | chr6:170272505..170272566:- | 5.4 | 78 +/- 3% | 18 | 18 | 0 | 0 | yes | sv40-miR-S1-5p |
| chr4_10490 | chr4:53486286..53486370:- | 5.4 | 78 +/- 3% | 240 | 240 | 0 | 0 | yes | sly-miR9473-5p |
| chr8_18808 | chr8:143406732..143406779:+ | 5.4 | 78 +/- 3% | 15 | 15 | 0 | 0 | yes | rlcv-miR-rL1-13-5p |
| chr1_1517 | chr1:212415059..212415113:+ | 5.4 | 78 +/- 3% | 75 | 75 | 0 | 0 | yes | zma-miR408b-5p |
| chr2_3659 | chr2:9739612..9739667:+ | 5.4 | 78 +/- 3% | 93 | 93 | 0 | 0 | yes | gga-miR-122b |
| chr7_16795 | chr7:157567453..157567494:+ | 5.4 | 78 +/- 3% | 214 | 214 | 0 | 0 | yes | mmu-miR-3473c |
| chr8_18670 | chr8:124851664..124851724:+ | 5.4 | 78 +/- 3% | 31 | 31 | 0 | 0 | yes | dps-miR-210b |
| chr8_19758 | chr8:141371845..141371908:- | 5.3 | 78 +/- 3% | 148 | 148 | 0 | 0 | yes | ath-miR853 |
| chrX_39852 | chrX:104013020..104013063:+ | 5.3 | 78 +/- 3% | 299 | 299 | 0 | 0 | yes | osa-miR528-5p |
| chr1_380 | chr1:30657188..30657244:+ | 5.3 | 78 +/- 3% | 236 | 236 | 0 | 0 | yes | sbi-miR6232a-3p |
| chr1_726 | chr1:77812368..77812422:+ | 5.3 | 78 +/- 3% | 204 | 204 | 0 | 0 | yes | cel-let-7-5p |
| chr11_24417 | chr11:116819736..116819781:+ | 5.3 | 78 +/- 3% | 16 | 16 | 0 | 0 | yes | csi-miR3952 |
| chr12_26780 | chr12:48827654..48827719:- | 5.3 | 78 +/- 3% | 633 | 633 | 0 | 0 | yes | zma-miR319a-5p |
| chr9_21403 | chr9:126900555..126900631:- | 5.3 | 78 +/- 3% | 24 | 22 | 1 | 1 | yes | cfa-miR-8892 |
| chr1_3473 | chr1:233695492..233695561:- | 5.3 | 78 +/- 3% | 19 | 19 | 0 | 0 | yes | dre-miR-456 |
| chr2_3834 | chr2:31522248..31522287:+ | 5.3 | 78 +/- 3% | 409 | 409 | 0 | 0 | yes | smo-miR1109 |
| chr8_18637 | chr8:121170416..121170463:+ | 5.3 | 78 +/- 3% | 5 | 2 | 0 | 3 | yes | odi-miR-1482 |
| chr1_3586 | chr1:247911332..247911414:- | 5.3 | 78 +/- 3% | 181 | 181 | 0 | 0 | yes | bmo-miR-14-5p |
| chr6_13843 | chr6:39074311..39074383:+ | 5.3 | 78 +/- 3% | 208 | 208 | 0 | 0 | yes | mmu-miR-129-5p |
| chr14_29534 | chr14:67675322..67675380:- | 5.3 | 78 +/- 3% | 317 | 317 | 0 | 0 | yes | ppy-miR-4451 |
| chr2_4880 | chr2:209772005..209772053:+ | 5.3 | 78 +/- 3% | 458 | 458 | 0 | 0 | yes | mmu-miR-3960 |
| chr19_35657 | chr19:38100154..38100217:+ | 5.3 | 78 +/- 3% | 68 | 68 | 0 | 0 | yes | eca-miR-9003 |
| chr9_21395 | chr9:125215362..125215427:- | 5.3 | 78 +/- 3% | 422 | 422 | 0 | 0 | yes | cel-let-7-5p |
| chr4_10045 | chr4:160384844..160384905:+ | 5.3 | 78 +/- 3% | 63 | 63 | 0 | 0 | yes | cin-miR-4116-5p |
| chr11_24433 | chr11:119492544..119492594:+ | 5.3 | 78 +/- 3% | 271 | 271 | 0 | 0 | yes | mdo-miR-7393-3p |
| chrX_40183 | chrX:1252941..1253012:- | 5.3 | 78 +/- 3% | 21 | 21 | 0 | 0 | yes | cin-miR-4065-5p |
| chr14_29842 | chr14:103605561..103605631:- | 5.3 | 78 +/- 3% | 110 | 105 | 5 | 0 | yes | zma-miR399d-5p |
| chr11_25097 | chr11:72673954..72674004:- | 5.3 | 78 +/- 3% | 23 | 23 | 0 | 0 | yes | gga-miR-6547-3p |
| chr5_11248 | chr5:10461978..10462037:+ | 5.3 | 78 +/- 3% | 2932 | 2932 | 0 | 0 | yes | bta-miR-2331-5p |
| chr19_35397 | chr19:11350811..11350872:+ | 5.3 | 78 +/- 3% | 21 | 21 | 0 | 0 | yes | ptr-miR-3151 |
| chr22_38610 | chr22:34804760..34804802:+ | 5.3 | 78 +/- 3% | 27 | 27 | 0 | 0 | yes | dps-miR-2522b-3p |
| chr15_30465 | chr15:25059880..25059947:- | 5.3 | 78 +/- 3% | 36 | 36 | 0 | 0 | yes | mcmv-miR-m21-1 |
| chr1_1742 | chr1:237603313..237603397:+ | 5.3 | 78 +/- 3% | 605 | 601 | 0 | 4 | yes | osa-miR1876 |
| chr9_21392 | chr9:124858771..124858814:- | 5.3 | 78 +/- 3% | 561 | 561 | 0 | 0 | yes | mmu-miR-677-3p |
| chr11_24505 | chr11:127412854..127412889:+ | 5.3 | 78 +/- 3% | 34 | 34 | 0 | 0 | yes | ssc-miR-4331 |
| chr16_31743 | chr16:79291248..79291329:+ | 5.3 | 78 +/- 3% | 143 | 143 | 0 | 0 | yes | zma-miR408b-5p |
| chr19_36451 | chr19:46565230..46565288:- | 5.3 | 78 +/- 3% | 584 | 584 | 0 | 0 | yes | gga-miR-1458 |
| chr2_4927 | chr2:218261930..218261988:+ | 5.3 | 78 +/- 3% | 37 | 37 | 0 | 0 | yes | gga-miR-1708 |
| chr1_2831 | chr1:153245725..153245764:- | 5.3 | 78 +/- 3% | 71 | 71 | 0 | 0 | yes | zma-miR399d-5p |
| chrY_41149 | chrY:1252941..1253012:- | 5.3 | 78 +/- 3% | 21 | 21 | 0 | 0 | yes | cin-miR-4065-5p |
| chr17_34146 | chr17:78129153..78129213:- | 5.3 | 78 +/- 3% | 908 | 908 | 0 | 0 | yes | cel-miR-271 |
| chr2_6073 | chr2:131659110..131659198:- | 5.3 | 78 +/- 3% | 230 | 230 | 0 | 0 | yes | mmu-miR-744-5p |
| chr14_29325 | chr14:28767418..28767485:- | 5.3 | 78 +/- 3% | 143 | 121 | 22 | 0 | yes | mmu-miR-3960 |
| chr9_21477 | chr9:133238434..133238487:- | 5.3 | 78 +/- 3% | 155 | 155 | 0 | 0 | yes | mmu-miR-3968 |
| chr18_34397 | chr18:21970173..21970239:+ | 5.3 | 78 +/- 3% | 29 | 29 | 0 | 0 | yes | oan-miR-1419c-5p |
| chr19_36519 | chr19:49954299..49954385:- | 5.3 | 78 +/- 3% | 264 | 264 | 0 | 0 | yes | gma-miR5766 |
| chr17_34167 | chr17:79099497..79099550:- | 5.3 | 78 +/- 3% | 61 | 61 | 0 | 0 | yes | ptr-miR-1225 |
| chr19_35430 | chr19:13140636..13140710:+ | 5.3 | 78 +/- 3% | 144 | 144 | 0 | 0 | yes | ath-miR164a |
| chr17_33937 | chr17:50866985..50867041:- | 5.2 | 78 +/- 3% | 30 | 30 | 0 | 0 | yes | cin-miR-4040-5p |
| chr7_16873 | chr7:2543391..2543432:- | 5.2 | 78 +/- 3% | 96 | 96 | 0 | 0 | yes | gma-miR1517 |
| chr7_16870 | chr7:2424792..2424863:- | 5.2 | 78 +/- 3% | 39 | 39 | 0 | 0 | yes | gga-miR-1813 |
| chr17_33124 | chr17:50363698..50363736:+ | 5.2 | 78 +/- 3% | 110 | 110 | 0 | 0 | yes | gga-miR-1607 |
| chr9_21377 | chr9:124100957..124101011:- | 5.2 | 78 +/- 3% | 682 | 682 | 0 | 0 | yes | mml-miR-134-3p |
| chr21_38314 | chr21:43075981..43076027:- | 5.2 | 78 +/- 3% | 98 | 98 | 0 | 0 | yes | rlcv-miR-rL1-22-5p |
| chr15_30339 | chr15:88139072..88139124:+ | 5.2 | 78 +/- 3% | 39 | 39 | 0 | 0 | yes | rno-miR-339-5p |
| chr19_36190 | chr19:17668260..17668328:- | 5.2 | 78 +/- 3% | 32 | 32 | 0 | 0 | yes | ppy-miR-1538 |
| chr5_12667 | chr5:56688627..56688691:- | 5.2 | 78 +/- 3% | 457 | 457 | 0 | 0 | yes | mmu-miR-146a-5p |
| chr3_7188 | chr3:59559769..59559818:+ | 5.2 | 78 +/- 3% | 12 | 10 | 0 | 2 | no | mmu-miR-881-3p |
| chr9_21559 | chr9:137844469..137844525:- | 5.2 | 78 +/- 3% | 152 | 152 | 0 | 0 | yes | bta-miR-2285t |
| chrX_40053 | chrX:140088059..140088108:+ | 5.2 | 78 +/- 3% | 287 | 287 | 0 | 0 | yes | cel-miR-37-5p |
| chr8_19578 | chr8:120720213..120720273:- | 5.2 | 78 +/- 3% | 437 | 437 | 0 | 0 | yes | mmu-miR-6938-3p |
| chr9_20708 | chr9:137056118..137056196:+ | 5.2 | 78 +/- 3% | 11 | 11 | 0 | 0 | yes | mmu-miR-6991-5p |
| chr14_29739 | chr14:96964348..96964395:- | 5.2 | 78 +/- 3% | 1790 | 1790 | 0 | 0 | yes | gga-miR-1652 |
| chr18_34489 | chr18:37447519..37447568:+ | 5.2 | 78 +/- 3% | 1163 | 1163 | 0 | 0 | yes | gga-miR-1556 |
| chr7_16600 | chr7:134530418..134530456:+ | 5.2 | 78 +/- 3% | 33 | 33 | 0 | 0 | yes | hbv-miR-B20-5p |
| chr1_1797 | chr1:247424509..247424565:+ | 5.2 | 78 +/- 3% | 234 | 234 | 0 | 0 | yes | mmu-miR-3471 |
| chr15_30463 | chr15:24675846..24675915:- | 5.2 | 78 +/- 3% | 2102 | 2102 | 0 | 0 | yes | nvi-miR-2796 |
| chr21_38132 | chr21:6467659..6467705:- | 5.2 | 78 +/- 3% | 98 | 98 | 0 | 0 | yes | rlcv-miR-rL1-22-5p |
| chrX_39748 | chrX:75568005..75568070:+ | 5.2 | 78 +/- 3% | 151 | 151 | 0 | 0 | yes | bta-miR-2463 |
| chr11_23607 | chr11:3092257..3092304:+ | 5.2 | 78 +/- 3% | 14 | 14 | 0 | 0 | yes | mmu-miR-1958 |
| chr15_30239 | chr15:78039479..78039528:+ | 5.2 | 78 +/- 3% | 261 | 261 | 0 | 0 | yes | osa-miR5800 |
| chr1_1495 | chr1:209801267..209801335:+ | 5.2 | 78 +/- 3% | 50 | 50 | 0 | 0 | yes | sma-miR-8421-5p |
| chr11_24932 | chr11:57791447..57791511:- | 5.2 | 78 +/- 3% | 1946 | 1946 | 0 | 0 | yes | ppt-miR171a |
| chr16_31169 | chr16:2183122..2183190:+ | 5.1 | 78 +/- 3% | 296 | 296 | 0 | 0 | yes | dme-miR-4976-3p |
| chr22_39093 | chr22:37097615..37097672:- | 5.1 | 78 +/- 3% | 299 | 299 | 0 | 0 | yes | tae-miR1124 |
| chrX_39525 | chrX:41182285..41182324:+ | 5.1 | 78 +/- 3% | 35 | 35 | 0 | 0 | yes | sbi-miR6233-5p |
| chr1_2267 | chr1:42241309..42241376:- | 5.1 | 78 +/- 3% | 12 | 12 | 0 | 0 | yes | pxy-miR-2756 |
| chr20_37227 | chr20:5382446..5382496:- | 5.1 | 78 +/- 3% | 111 | 111 | 0 | 0 | yes | sma-miR-8443-3p |
| chr1_2315 | chr1:48033375..48033418:- | 5.1 | 78 +/- 3% | 93 | 93 | 0 | 0 | yes | bta-miR-2319b |
| chr13_27522 | chr13:35763831..35763900:+ | 5.1 | 78 +/- 3% | 18 | 18 | 0 | 0 | yes | mmu-miR-183-5p |
| chr17_33921 | chr17:49226804..49226873:- | 5.1 | 78 +/- 3% | 87 | 87 | 0 | 0 | yes | mmu-miR-504-5p |
| chr10_23501 | chr10:131116310..131116373:- | 5.1 | 78 +/- 3% | 114 | 114 | 0 | 0 | yes | gga-miR-1458 |
| chr10_23047 | chr10:80252821..80252898:- | 5.1 | 78 +/- 3% | 1941 | 1652 | 289 | 0 | yes | oan-miR-1421ac-5p |
| chr2_3833 | chr2:31522169..31522241:+ | 5.1 | 78 +/- 3% | 13 | 13 | 0 | 0 | yes | mmu-miR-7010-5p |
| chrX_39459 | chrX:25039334..25039399:+ | 5.1 | 78 +/- 3% | 87 | 87 | 0 | 0 | yes | gga-miR-1791-5p |
| chr3_8344 | chr3:52025062..52025124:- | 5.1 | 78 +/- 3% | 17 | 17 | 0 | 0 | yes | gga-miR-6581-5p |
| chr7_17238 | chr7:65238455..65238520:- | 5.1 | 78 +/- 3% | 91 | 91 | 0 | 0 | yes | dme-miR-954-5p |
| chr11_24620 | chr11:2863300..2863344:- | 5.1 | 78 +/- 3% | 12 | 12 | 0 | 0 | yes | mmu-miR-709 |
| chr11_25056 | chr11:67698410..67698448:- | 5.1 | 78 +/- 3% | 89 | 89 | 0 | 0 | yes | ath-miR159b-5p |
| chr18_34824 | chr18:9766165..9766232:- | 5.1 | 78 +/- 3% | 25 | 25 | 0 | 0 | yes | osa-miR319a-3p |
| chr13_28200 | chr13:53145011..53145092:- | 5.1 | 78 +/- 3% | 180 | 180 | 0 | 0 | yes | eca-miR-9131 |
| chrX_40520 | chrX:68694982..68695069:- | 5.1 | 78 +/- 3% | 23 | 23 | 0 | 0 | yes | gga-miR-6547-3p |
| chr2_5448 | chr2:35919124..35919186:- | 5.1 | 78 +/- 3% | 15 | 15 | 0 | 0 | yes | osa-miR812v |
| chr2_4382 | chr2:113769525..113769601:+ | 5.1 | 78 +/- 3% | 50 | 50 | 0 | 0 | yes | ppe-miR6294 |
| chr4_10407 | chr4:35826952..35827028:- | 5.1 | 78 +/- 3% | 152 | 148 | 4 | 0 | yes | cel-miR-37-5p |
| chr16_31572 | chr16:57095189..57095251:+ | 5.1 | 78 +/- 3% | 41 | 41 | 0 | 0 | yes | gga-miR-1813 |
| chr1_1692 | chr1:230415494..230415559:+ | 5.1 | 78 +/- 3% | 673 | 673 | 0 | 0 | yes | rno-miR-100-3p |
| chr22_39152 | chr22:41303121..41303182:- | 5.1 | 78 +/- 3% | 1077 | 1077 | 0 | 0 | yes | mmu-miR-3092-5p |
| chr15_30178 | chr15:68428379..68428447:+ | 5 | 78 +/- 3% | 114 | 114 | 0 | 0 | yes | mtr-miR5563-3p |
| chr19_36150 | chr19:14337679..14337750:- | 5 | 78 +/- 3% | 29 | 29 | 0 | 0 | yes | mmu-miR-7658-5p |
| chr7_16031 | chr7:30430995..30431079:+ | 5 | 78 +/- 3% | 215 | 215 | 0 | 0 | yes | mmu-miR-365-3p |
| chr1_245 | chr1:18874094..18874142:+ | 5 | 78 +/- 3% | 28 | 28 | 0 | 0 | yes | ppc-miR-8357-3p |
| chr19_35704 | chr19:40543522..40543589:+ | 5 | 78 +/- 3% | 134 | 134 | 0 | 0 | yes | mdv2-miR-M25-3p |
| chr6_15607 | chr6:146596044..146596087:- | 5 | 78 +/- 3% | 266 | 266 | 0 | 0 | yes | ptc-miR477a-5p |
| chrX_40995 | chrX:155477879..155477955:- | 5 | 78 +/- 3% | 147 | 147 | 0 | 0 | yes | mmu-miR-28a-5p |
| chr6_13747 | chr6:31791852..31791903:+ | 5 | 78 +/- 3% | 27 | 27 | 0 | 0 | yes | iltv-miR-I5-3p |
| chr3_8233 | chr3:42536654..42536711:- | 5 | 78 +/- 3% | 441 | 441 | 0 | 0 | yes | smo-miR1107 |
| chr17_33567 | chr17:8131832..8131871:- | 5 | 78 +/- 3% | 77 | 77 | 0 | 0 | yes | vvi-miR171h |
| chr4_10508 | chr4:56398886..56398940:- | 5 | 78 +/- 3% | 138 | 138 | 0 | 0 | yes | cel-let-7-5p |
| chr5_12647 | chr5:54703375..54703438:- | 5 | 78 +/- 3% | 43 | 43 | 0 | 0 | yes | cre-miR1160.1 |
| chr7_16517 | chr7:120598519..120598590:+ | 5 | 78 +/- 3% | 34 | 34 | 0 | 0 | yes | bta-miR-2327 |
| chr20_36704 | chr20:16364501..16364571:+ | 5 | 78 +/- 3% | 5727 | 5727 | 0 | 0 | yes | cin-miR-4017-5p |
| chr17_32901 | chr17:33228828..33228908:+ | 5 | 78 +/- 3% | 80 | 80 | 0 | 0 | yes | rno-miR-339-5p |
| chr1_3036 | chr1:170894206..170894268:- | 5 | 78 +/- 3% | 160 | 160 | 0 | 0 | yes | dvi-miR-9545-5p |
| chr2_4650 | chr2:160657427..160657482:+ | 5 | 78 +/- 3% | 19 | 19 | 0 | 0 | yes | dme-miR-190-3p |
| chrX_40769 | chrX:123844348..123844385:- | 5 | 78 +/- 3% | 1446 | 1446 | 0 | 0 | yes | esi-miR3466-5p |
| chr15_30480 | chr15:27599615..27599659:- | 5 | 78 +/- 3% | 232 | 232 | 0 | 0 | yes | mmu-miR-5122 |
| chr14_29144 | chr14:100349652..100349709:+ | 5 | 78 +/- 3% | 11 | 11 | 0 | 0 | yes | mmu-miR-326-5p |
| chr12_26570 | chr12:7681491..7681574:- | 5 | 78 +/- 3% | 5824 | 5824 | 0 | 0 | yes | tgu-miR-7645-3p |
| chr22_38777 | chr22:46915034..46915085:+ | 5 | 78 +/- 3% | 49 | 49 | 0 | 0 | yes | mml-miR-30b-3p |
| chr1_3408 | chr1:226891732..226891813:- | 5 | 78 +/- 3% | 112 | 112 | 0 | 0 | yes | gsa-miR-71b-3p |
| chr10_22395 | chr10:119003845..119003911:+ | 5 | 78 +/- 3% | 234 | 234 | 0 | 0 | yes | mml-miR-7175-5p |
| chr2_5595 | chr2:64990732..64990813:- | 5 | 78 +/- 3% | 412 | 412 | 0 | 0 | yes | tur-miR-5731-5p |
| chr8_18882 | chr8:6475623..6475685:- | 5 | 78 +/- 3% | 142 | 142 | 0 | 0 | yes | tgu-miR-92-2-5p |
| chr3_7047 | chr3:42754041..42754098:+ | 5 | 78 +/- 3% | 176 | 176 | 0 | 0 | yes | prd-miR-35a-5p |
| chr17_33471 | chr17:352529..352580:- | 5 | 78 +/- 3% | 28 | 28 | 0 | 0 | yes | ptr-miR-3192 |
| chr3_8808 | chr3:138905305..138905346:- | 5 | 78 +/- 3% | 204 | 204 | 0 | 0 | yes | cel-let-7-5p |
| chr13_27878 | chr13:106546989..106547045:+ | 5 | 78 +/- 3% | 13 | 13 | 0 | 0 | yes | osa-miR5543 |

**Figure S1 Characterization of exosomes derived from human breast milk**


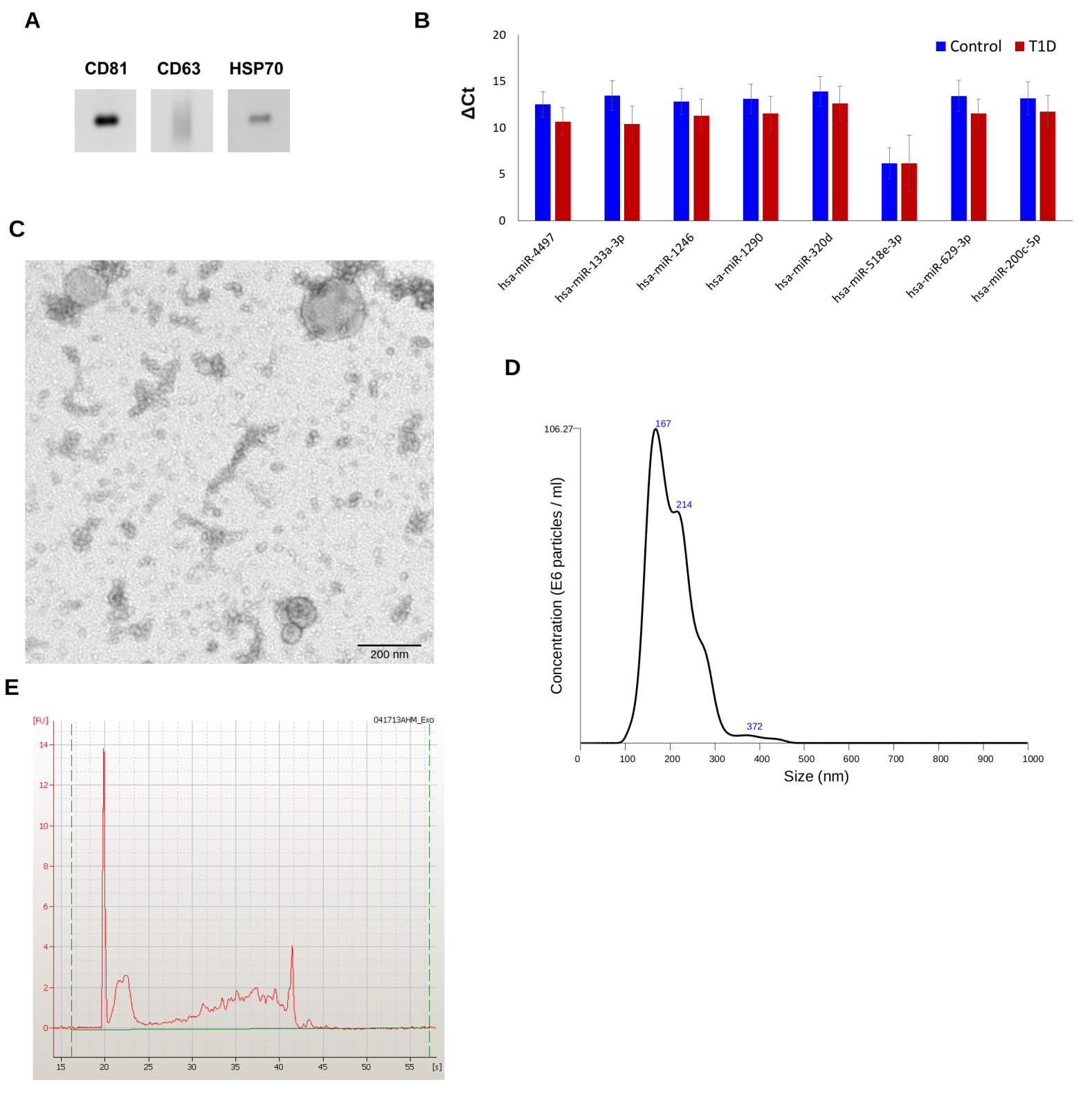


(A) Exosomal surface markers. CD81 antibody: Rabbit polyclonal IgG (1:1000 dilution); CD63 antibody: Rabbit polyclonal IgG , (1:500 dilution) Santa Cruz Biotechnology; HSP70 antibody: Rabbit polyclonal (Cell Signaling)1:1000 dilution). (B) Relative expression of 8 differentially expressed miRNAs as measured by RT-qPCR in four T1D patients and four healthy controls. Bars represent mean values of delta Ct per sample category, normalized to hsa-miR-7a-5p. (C) Electron micrograph of breast milk derived exosomes negatively stained with 1% uranyl acetate using Phillips CM100(a) electron microscope. (D) Exosomal size distribution by Nanosight NTA analysis. (E) Analysis of Exosomal RNA by Bioanalyzer.

Full western blot image of exosomal surface markers

**
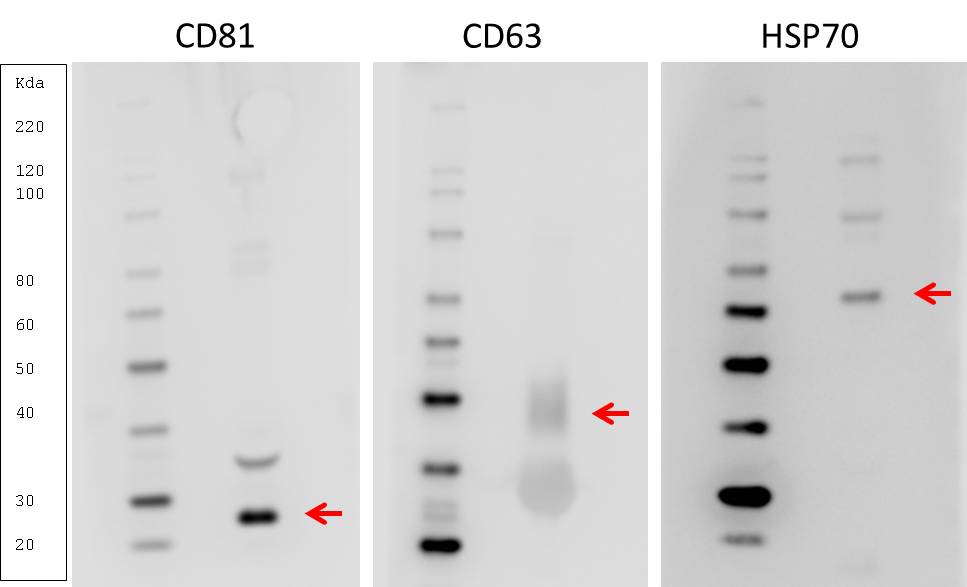
**

**Figure S2 Library size and total number of miRNAs**


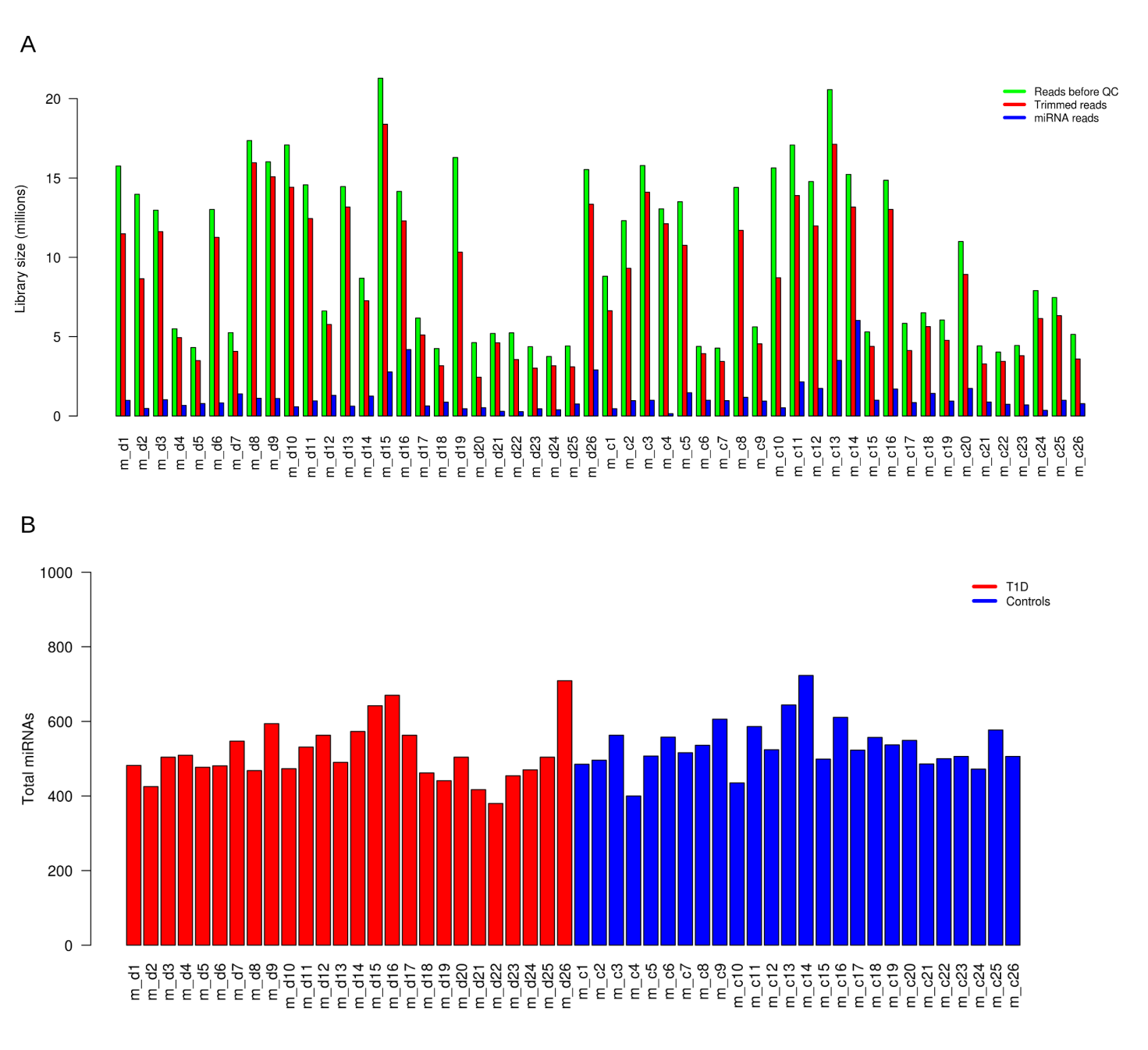


(A) Distribution of reads after QC and number of reads mapping to miRNAs (B) Total number of miRNAs identified in breast milk samples from T1D and control mothers.

**Figure S3: Target genes for the up-regulated miRNAs**

**
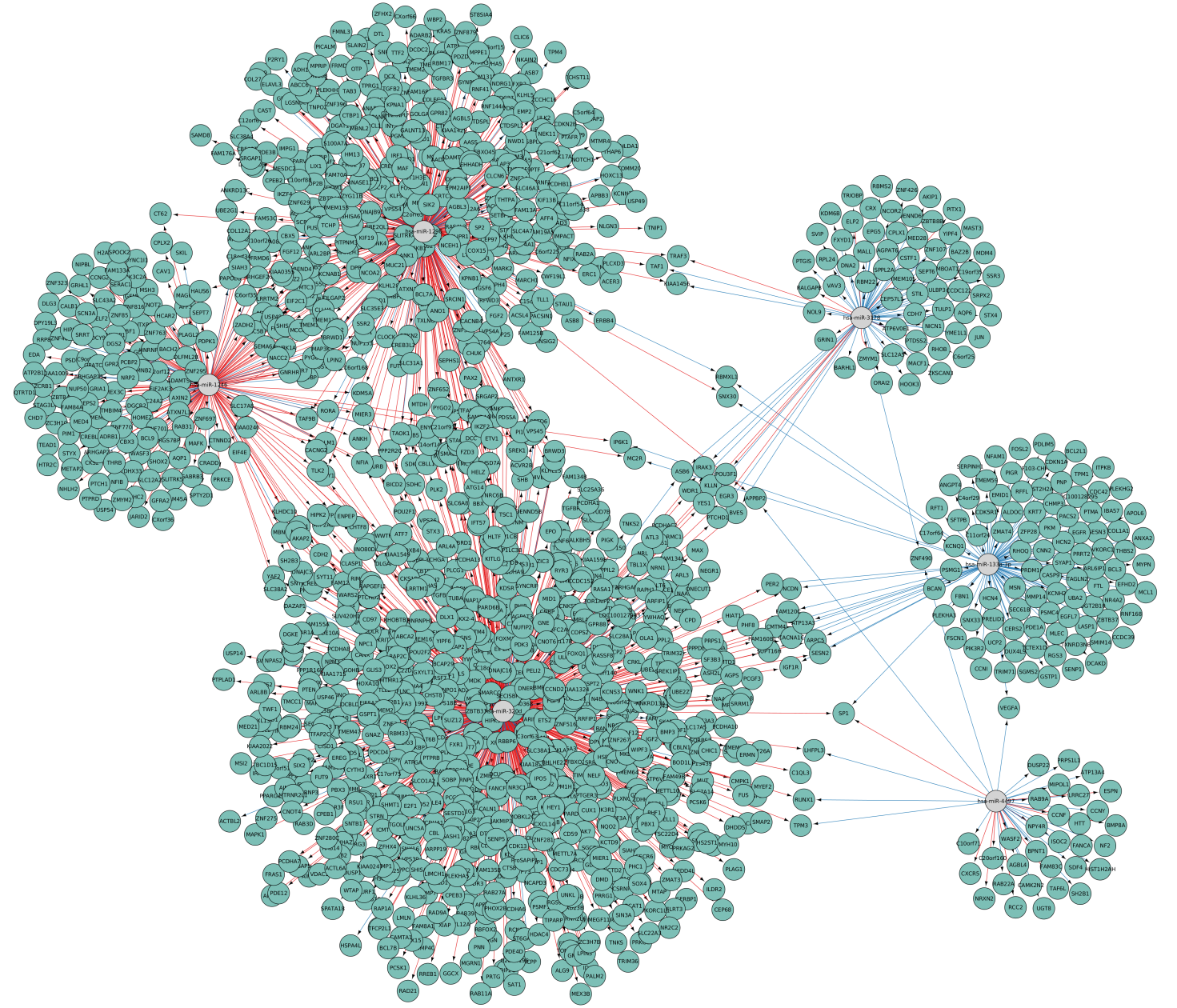
**

Predicted and experimentally validated target genes retrieved from TargetScan (red edges) and miRTarBase (blue edges) are shown for the 6 up-regulated miRNAs. The nodes in grey represent the miRNAs and the nodes in green represent the target genes.

**Figure S4: Target genes for the down-regulated miRNAs**

**
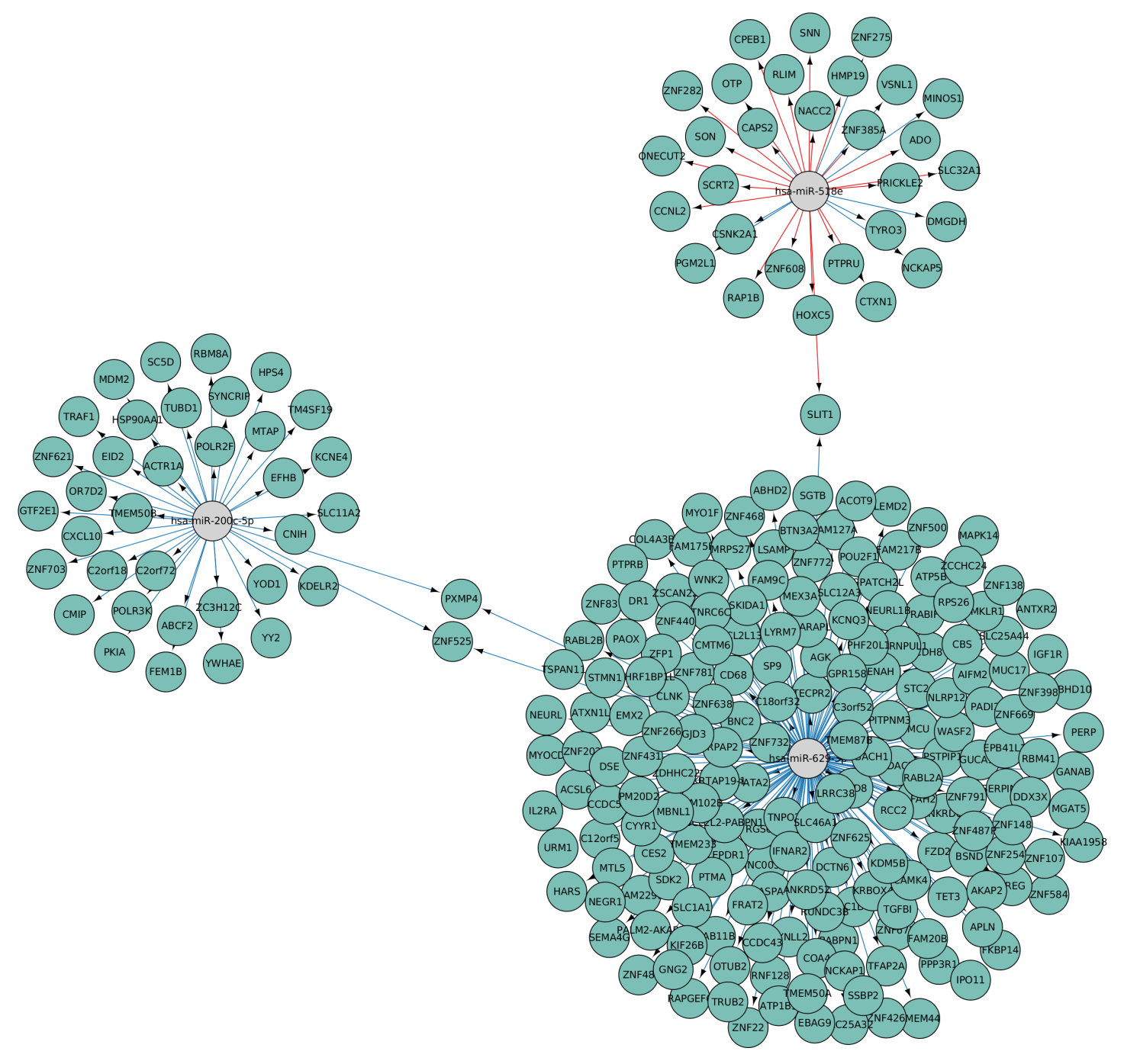
**

Predicted and experimentally validated target genes retrieved from TargetScan (red arrows) and miRTarBase (blue arrows) are shown for the 3 up-regulated miRNAs. The nodes in grey represent the miRNAs and the nodes in green represent the target genes.

**Figure S5:**

**
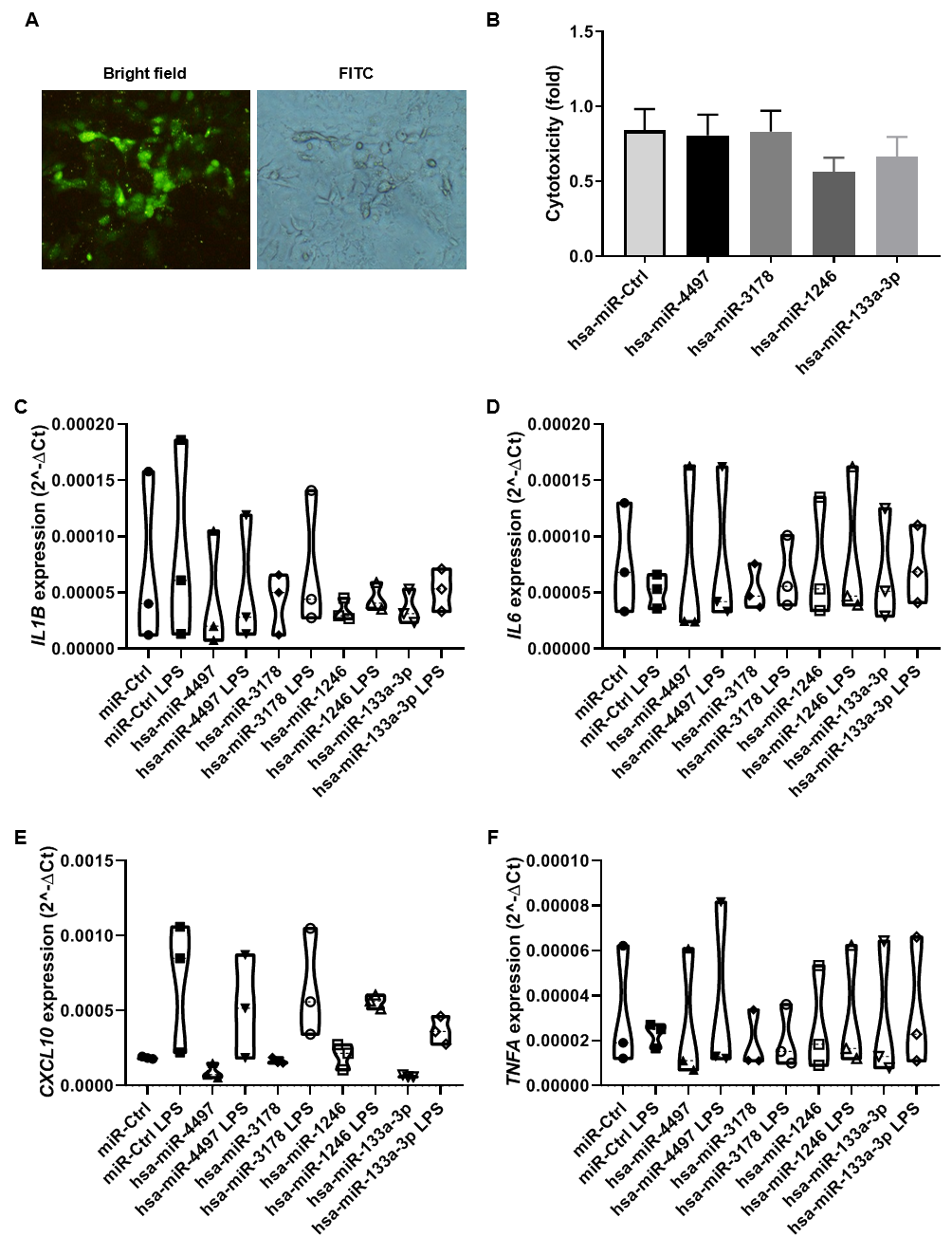
**

(A) Fluorescence microscopy of human epithelial CaCo-2 cells transfected with siGLO. (B) CaCo-2 cells transfected with negative control miRNA (miR-Ctrl) or hsa-miR-4497, hsa-miR-1246, hsa-miR-133a-3p and hsa-miR-3178 were subject to cytotoxicity assay two days post transfection to determine cell viability. Data are means ± SEM of n=3. (C–F) Gene expression in CaCo-2 cells transfected as in (B) and exposed to 5 ng/ml LPS for 3 h was measured by realtime PCR and normalized to that of *ACTB*. Data are means ± SEM of n=3 (A-F).

**Figure S6: ANCOVA regression analysis of up-regulated miRNAs and HbA1c levels**

**
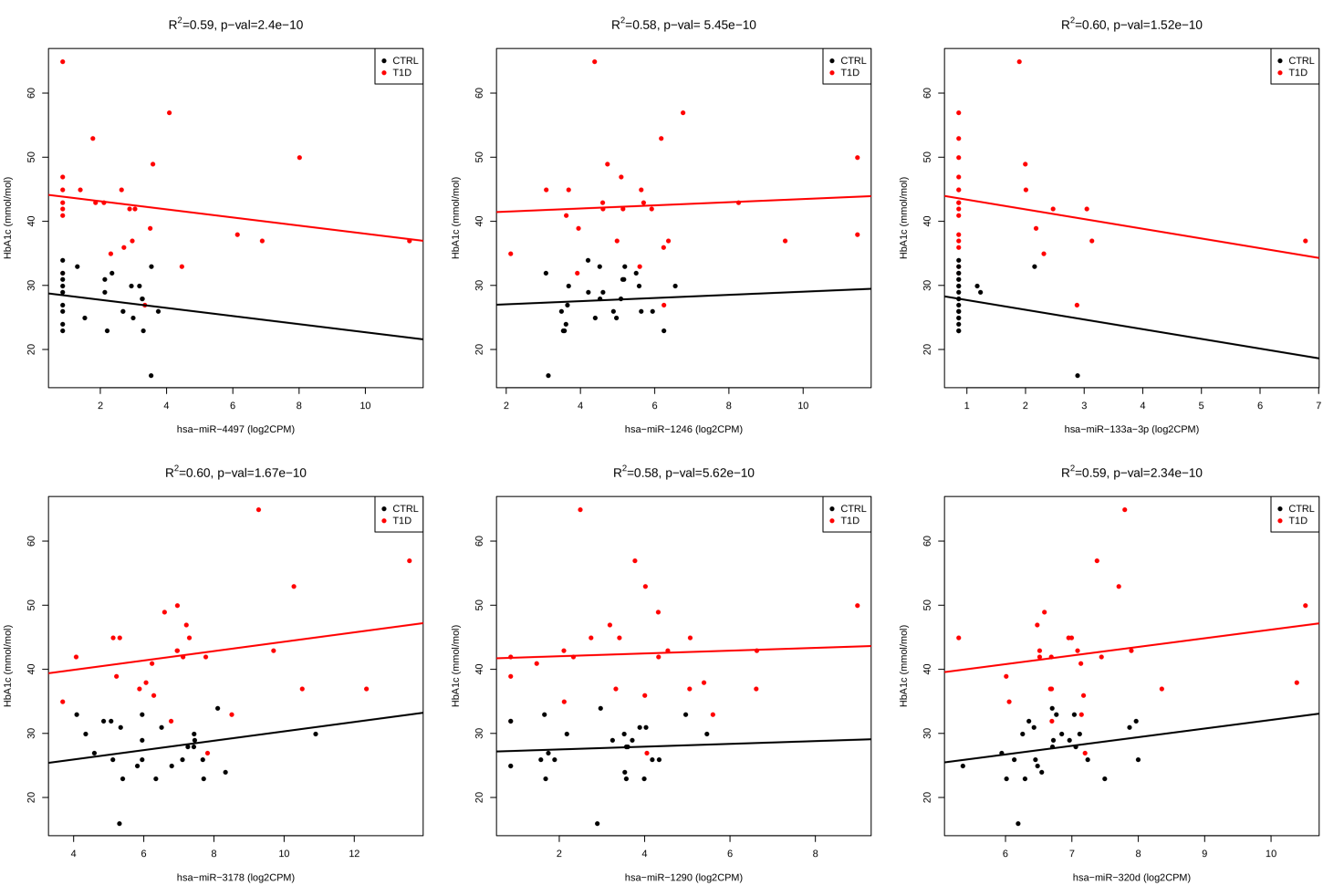
**

**Figure S7: ANCOVA regression analysis of down-regulated miRNAs and HbA1c levels**

**
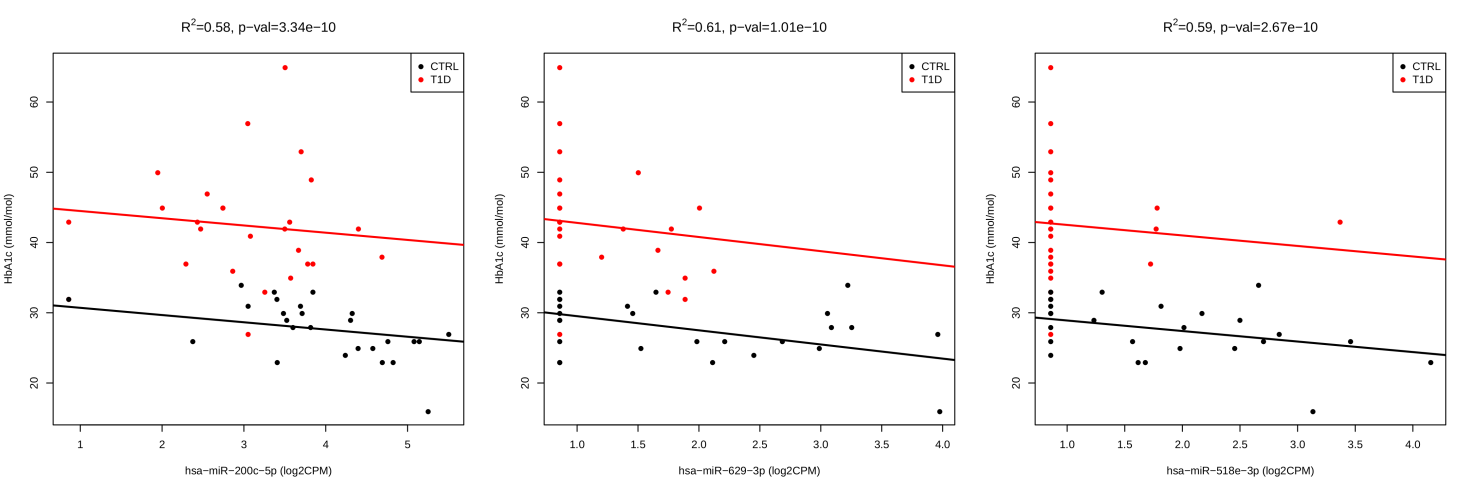
**
